# The application of a biostimulant based on tannins affects root architecture and improves tolerance to salinity in tomato plants

**DOI:** 10.1038/s41598-020-79770-5

**Published:** 2021-01-11

**Authors:** Cristina Campobenedetto, Giuseppe Mannino, Jules Beekwilder, Valeria Contartese, Rumyana Karlova, Cinzia M. Bertea

**Affiliations:** 1 Plant Physiology Unit, Department of Life Sciences and Systems Biology, University of Torino, Turin, Italy; 2 Green Has Italia S.P.A, Canale, CN Italy; 3 Wageningen University and Research Centre, Bioscience, Wageningen, The Netherlands; 4 Laboratory of Plant Physiology, Plant Sciences Group, Wageningen University and Research, 6708 PB 9 Wageningen, The Netherlands

**Keywords:** Transcriptomics, Plant sciences, Mass spectrometry

## Abstract

Roots have important roles for plants to withstand adverse environmental conditions, including salt stress. Biostimulant application was shown to enhance plant resilience towards abiotic stresses. Here, we studied the effect of a tannin-based biostimulant on tomato (*Solanum lycopersicum* L.) grown under salt stress conditions. We investigated the related changes at both root architecture (via imaging and biometric analysis) and gene expression (RNA-Seq/qPCR) levels. Moreover, in order to identify the main compounds potentially involved in the observed effects, the chemical composition of the biostimulant was evaluated by UV/Vis and HPLC-ESI-Orbitrap analysis. Sixteen compounds, known to be involved in root development and having a potential antioxidant properties were identified. Significant increase of root weight (+ 24%) and length (+ 23%) was observed when the plants were grown under salt stress and treated with the biostimulant. Moreover, transcriptome analysis revealed that the application of the biostimulant upregulated 285 genes, most of which correlated to root development and salt stress tolerance. The 171 downregulated genes were mainly involved in nutrient uptake. These data demonstrated that the biostimulant is able not only to restore root growth in salty soils, but also to provide the adequate plant nourishment by regulating the expression of essential transcription factors and stress responsive genes.

## Introduction

In the last years, due to the increasing of human population and the decreasing of arable lands^1–3^, new and more sustainable agriculture practices have to be employed to guarantee an higher crop yield. Moreover, the production of higher quality food, with a low environmental impact, is also one of the main important challenges of the modern agriculture^4^. Indeed, the reduction of pesticides, the losses of nutrients in the soil, the optimal use of water and nutrients, as well as the preservation of soil fertility, are fundamental aspects to take into consideration^5^.

In order to enhance the production standards by maintaining at the same time food safety and quality, scientists have been working for years to improve plant traits. Some of these traits are directly related to the final yield, such as number of flowers and fruits, nutritional content and appearance. Other parameters, for example those linked to root development, have an indirect effect on crop yield and value, and rather aim at resistance against biotic and abiotic stress. These effects are due to the involvement of roots in several processes, including the uptake of nutrients and water, the interactions with soil microorganisms, and establishment of the plant in the soil. Moreover, the root system is directly in contact with the surrounding environment, and thus responds early to different stresses, such as for example salinity^6^. Salt stress represents one of the most important limiting factors for crop production, affecting plant growth and development, and causing water leakage and nutritional disorders^7^. About 20% of arable lands worldwide is affected by salinity and this problem is expanding, due to climate change and its effect on water management in agriculture^8^.

In order to develop crops which are resilient to salt stress, new plant breeding techniques and practices need to be developed. Currently, helping the plants to survive stress at the early stress responses is an everyday-challenge for farmers. Among the new generation products available on the market, biostimulants could be used for this purpose. They are defined by the new Regulation (EU) 2019/1009 as “products stimulating plant nutrition processes independently of the product’s nutrient content with the sole aim of improving one or more of the following characteristics of the plant or the plant rhizosphere: nutrient use efficiency, tolerance to abiotic stress, quality traits, availability of confined nutrients in soil or rhizosphere”^9^. In general, biostimulants are not involved in a direct action against pathogens and biotic stress, but they are largely employed to enhance plant performance, especially under environmental adverse conditions.

In this study, a biostimulant based on a mixture of hydrolysable and condensed tannins derived from waste industry (VIVEMA TWIN) was tested on tomato (*Solanum lycopersicum* L.) plants under optimal and salt stress conditions. *S. lycopersicum* is an established crop model system with global economical relevance^10,11^. Moreover, the importance of this crop as food source, the easy greenhouse cultivation, and the large amount of data present in literature, make tomato a good crop model system.

Tannins are water-soluble and condensed phenolic compounds of variable size widely distributed in the plant kingdom^12^. In addition to their use in leather treatment, as textile dyes and coagulant agents in rubber production^13^, tannins have been also shown to have several applications in animal and human nutrition, due to their antioxidant properties and their ability to bind and precipitate proteins^14^. For these reasons they could be considered as a good green alternative not only in food and nutraceutical industry but also in other fields^15^. On the other hand, the use of tannins in agricultural practices and in plant nutrition is still poorly explored. Few studies on the effect of tannins on plant performance have been reported in literature^16^. In particular, it has been suggested that gallic acid (GA), a compound found in high quantity in tannin rich mixtures, has an effect on root development. Its effects were shown to be mainly related to its antioxidant and auxin-like activity^17^, and were reflected in an increase in growth and antioxidant defenses in gallic acid-treated roots of plants subjected to salt stress^18^.

In this work, the chemical composition of the biostimulant VIVEMA TWIN was first investigated by UV/Vis, and then via HPLC-ESI-Orbitrap MS analysis. In addition, the reducing and radical scavenging properties of the biostimulant were characterized spectrophotometrically. Finally, the mechanism of action of the biostimulant was studied in tomato at morphological and biomolecular level. Morphological estimation included the evaluation of the effect of VIVEMA TWIN on *S. lycopersicum* plants during short-term and long-term salt stress, by imaging analysis and monitoring of biometric parameters. The molecular targets of this innovative biostimulant were identified by RNA-Seq analysis, and the expression level of the most representative genes was confirmed by qPCR.

## Results and discussion

### HPLC-ESI-FTMS revealed that the main consistituents of VIVEMA TWIN are phenolic compounds belonging to the tannin family

Due to the different origin of biostimulants, their composition displays significant variations in the chemical profile, not only from a quantitative but mainly from a qualitative point of view^19^. Indeed, biostimulant composition may range from single compounds to complex combinations of bioactive components, only partly characterized^20^. However, the biological action of biostimulants on plants depends on the complexity of these matrices, and for this reason their characterization represents a challenge that needs to be addressed.

In this work, a preliminary spectrophotometric characterization of the VIVEMA TWIN biostimulant was carried out in order to perform an initial chemical screening useful for subsequent analyses with HPLC-ESI-FTMS. The spectrophotometric determination included the quantification of the total amount of polyphenols (TPC), anthocyanins (TAnthC) and flavan-3-ols (TF3C) estimated by Folin-Ciocalteu, pH differential and DMAC methods, respectively. Results of the UV/Vis quantification are shown in Table 1.

**Table 1:** UV/Vis spectrophotometric determination of bioactive compounds and antioxidant properties of VIVEMA TWIN.

Total polyphenol content	TPC	395.85 ± 19.07	mg GAE mL^−1^ biostimulant
Total Flavan-3-ol content	TF3C	174.42 ± 2.52	mg PAC-A mL^−1^ biostimulant
Total anthocyanin content	TAnthC	n.d	mg CyE mL^−1^ biostimulant
Radical scavenging activity	ABTS	790.95 ± 24.66	µmol TE mL^−1^ biostimulant
	DPPH	519.37 ± 11.43	µmol TE mL^−1^ biostimulant
Reducing activity	FRAP	688.71 ± 12.49	µmol TE mL^−1^ biostimulant

Total polyphenol content (TPC), total flavan-3-ol content (TF3C), total anthocyanin content (TAnthC), radical scavenging activity (ABTS and DPPH) and ferric reducing antioxidant power (FRAP) of VIVEMA TWIN. Values are expressed as a mean ± SD of three experiments carried out in triplicate.

*GAE* gallic acid equivalents, *PAC-A* A-type proanthocyanidin equivalent, *CyE* cyanidin-6-glucoside equivalents, *TE* trolox equivalents, *ABTS* 2,2′-azino-bis(3-ethylbenzothiazoline-6-sulfonic acid), *DPPH* 2,2-diphenyl-1-picrylhydrazyl, *FRAP* ferric reducing antioxidant power.

Interestingly, VIVEMA TWIN showed a very high TPC value when compared to the plant raw materials listed in the top 100 highly enriched in polyphenol content^21^. Polyphenols are characterized by the presence in their structure of one or more phenolic groups, able to accept electrons and reduce not only reactive oxygen species, but also organic substrates and minerals^22^. The strong redox properties of polyphenols explain the considerable interest in human and plant nutrition in order to prevent conditions associated with excessive oxidative stress^23^. Among polyphenols, several classes may be identified, mainly because of the close structural similarity and only few assays are able to detect and quantify these molecules selectively. In this context, the pH differential method is able to find the presence of anthocyanin compounds thanks to their characteristics to display different color depending of the pH of the environment mixture^14,24^. The 4-(Dimethylamino)-cinnamaldehyde (DMAC reagent) can selectively react with flavonol compounds with free meta‐oriented hydroxyl groups in the flavonoid scaffold, and with a single bond in 2,3‐position of the C‐ring^14,24,25^. The spectrophotometric determination obtained through these assays showed that, even though no anthocyanins were present (LOD: 3 μg mL^−1^; LOQ: 10 μg mL^−1^), almost 17% of the mixture consisted of flavan-3-ols.

**Figure 1:**
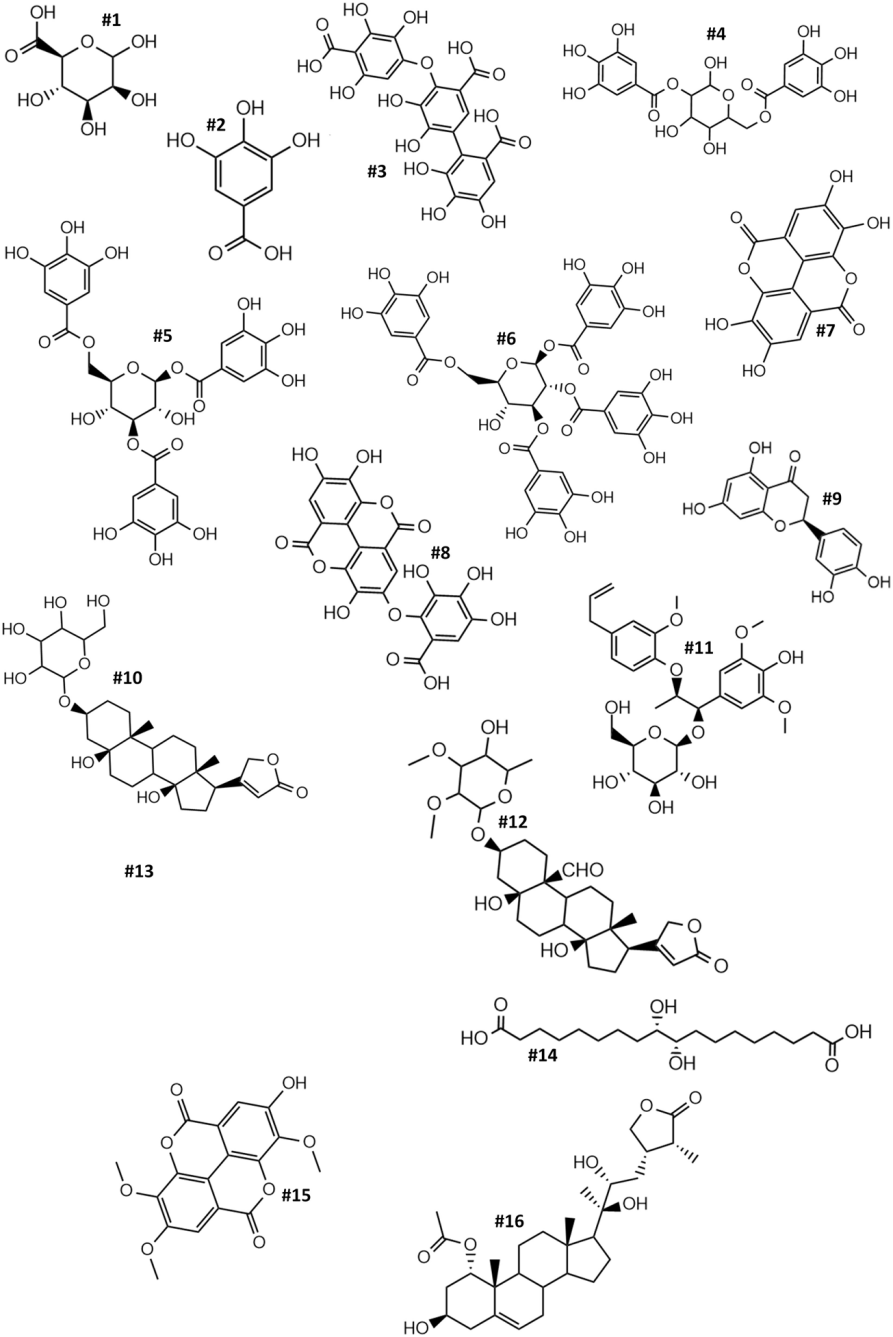
Chemical structures of the identified compounds in VIVEMA TWIN by HPLC-ESI-FTMS. Each chemical compound is reported in Table 2.

In order to identify the most important active compounds present in VIVEMA TWIN, HPLC-ESI-FTMS was employed. Based on both the fragmentation pattern of each compound and on their retention time, HPLC-ESI-FTMS analysis allowed the putative identification of 16 compounds (Fig. 1). Among the identified compounds, four are organic acid [Mannuronic Acid (**#1**), Gallic Acid (**#2**), Valoneic Acid (**#3**) and Phloionic Acid (**#14**)], three are condensed tannins derived from the condensation of GA (**#4**, **#5** and **#6**) and three are the lactone form of the **#3** [Ellagic Acid (**#7**), Valoneic acid (**#8**), and 2,3,8-Trimethylellagic Acid (**#15**)]. In particular, among the lactones, **#8** is a dilactone. Moreover, the HPLC-ESI-FTMS analysis identified other bioactive compounds, such as a caffeic acid derivatives (**#13**), one flavonoid (**#9**), one lignan (**#11**) and two natural steroids (**#12** and **#16**). The Molecular Weight (MW), Retention Time (RT), Molecular Formula and CAS ID of each compound is reported in Table 2. Most of the identified compounds are flavan-3-ols, and in particular simple and complex tannin building blocks of GA (**#2**, **#3**, **#4**, **#5**, **#6**, **#7**, **#8**, **#15**)^15^.

**Table 2:** Chemical characterization of VIVEMA TWIN by HPLC-ESI-FTMS analysis.

RT	MW	#	Compounds	Molecular formula	CAS ID
2.14	194.13	**1**	Mannuronic acid	C_6_H_10_O_7_	1986-14-7
4.57	170.12	**2**	Gallic acid	C_7_H_6_O_5_	149-91-7
10.81	474.32	**3**	Valoneic acid	C_21_H_14_O_13_	517-54-4
10.83	484.08	**4**	2,6-Digalloylglucose	C_20_H_20_O_14_	94356-20-4
16.27	636.32	**5**	1,3,6-Trigalloyl glucose	C_27_H_24_O_18_	18483-17-5
21.45	788.12	**6**	1,2,3,6-Tetra-*O*-galloyl-beta-d-glucose	C_34_H_28_O_22_	79886-50-3
22.24	302.19	**7**	Ellagic acid	C_14_H_6_O_8_	476-66-4
24.79	470.28	**8**	Valoneic acid dilactone	C_21_H_10_O_13_	60202-70-2
27.71	288.25	**9**	Eriodictyol	C_15_H_12_O_6_	552-58-9
32.26	552.71	**10**	Periplogenin-d-glucoside	C_29_H_44_O_10_	18531-44-7
36.11	566.62	**11**	Pharbilignoside	C_28_H_38_O_12_	N/A
39.09	726.38	**12**	Glucokamaloside	C_37_H_58_O_14_	N/A
40.92	536.41	**13**	1,6-Dicaffeoyl glucose	C_24_H_24_O_14_	56614-74-5
45.08	346.53	**14**	Phloionic acid	C_18_H_34_O_6_	23843-52-9
47.62	344.32	**15**	2,3,8-Tri-*O*-methylellagic acid	C_17_H_12_O_8_	1617-49-8
48.36	518.71	**16**	Perulactone	C_30_H_46_O_7_	76994-38-2

HPLC-ESI-FTMS analysis of the content of bioactive compounds found in the biostimulant VIVEMA TWIN. In the table retention time (RT), molecular weight (MW), molecular formula and CAS ID of each identified compound are reported.

N/A indicates not available CAS ID number.

The strong antioxidant properties of GA (**#2**), and its derivatives, have been reported in literature, and several studies claim the possible role and application of GA in plant nutrition at different stages of growth, especially in early root and plant development^26,27^. For example, Singh and co-workers showed that the application of exogenous GA on rice seeds led to an increase of root length compared to the control^27^. Moreover, Negi and colleagues showed that GA might have an auxin-like function, directly acting on root length and development^17^. Compound **#3** and the related monolactones (**#7** and **#15**) and dilactone (**#8**) are also hydrolysable tannins belonging to the family of ellagitannins^28^, known to exert an antioxidant activity^29^. Concerning condensed tannins, they are strong antioxidant compounds^30^, and due to this property are largely used to supplement human and animal diet^31^. However, the knowledge about the use of tannin-based products in agriculture is limited^16^ and need to be investigated. In addition, other bioactive compounds not belonging to the family of tannins were also found in the biostimulant. The compound **#9**, a flavone molecule, is known to be involved in root length development and in plant growth^32^. Compounds **#12** and **#16** correspond to natural steroids belonging to the triterpenoid family, which display interesting pharmacological effects on humans, modulating the Na^+^/K^+^-ATPase activity^33,34^. However, their potential action on plant physiology is still unknown. Finally, compounds **#11** and **#14**, are two natural substances derived from wood hydrolysis^35^.

### VIVEMA TWIN has antioxidant properties

In this work, the antioxidant properties of VIVEMA TWIN were investigated in term of both reducing and radical scavenging activity via FRAP, ABTS and DPPH assays. The data of these measurements are shown in Table 1. In general, all the assays displayed very high values, when compared to those reported in Phenol-Explorer database for the top ranking fruits and vegetables^21^. However, due to the lack of antioxidant evaluations previously carried out on biostimulants, it is not possible to make a comparison with products belonging to this category. The results shown here indicate a strong antioxidant activity of this biostimulant suggesting that this product could help plants to survive different stresses related to accumulation of Reactive Oxygen Species (ROS).

ROS play different roles in plants^36^, however their overproduction may result in undesirable consequence for the plants^37^. Indeed, once formed, ROS must be detoxified as efficiently as possible to minimize potential damage. Plant cells are protected by a complex antioxidant system, including both non-enzymatic and enzymatic defenses^38^. Plant polyphenolic compounds stored by plants in their fruits, flowers and leaves belong to the first category^39^. In this context, plant-based biostimulants originating from industrial wastes might be rich in polyphenols that, if applied to plants, may promote beneficial effects by reducing potential oxidative threats.

Currently, the evaluation of the antioxidant properties of fruit and vegetable extracts is a crucial point to better understand the action mechanism of plant-based supplements to be used for human and animal nutrition^40^. Conversely, although a large part of biostimulants are derived from plants, this approach is still not used for plant nutrition. On the other hand, the measurements of antioxidant parameters of plant biostimulants will help to understand their mechanism of action, and contribute for their optimal use in agriculture.

### VIVEMA TWIN and gallic acid are able to modify the root architecture in tomato

**Figure 2:**
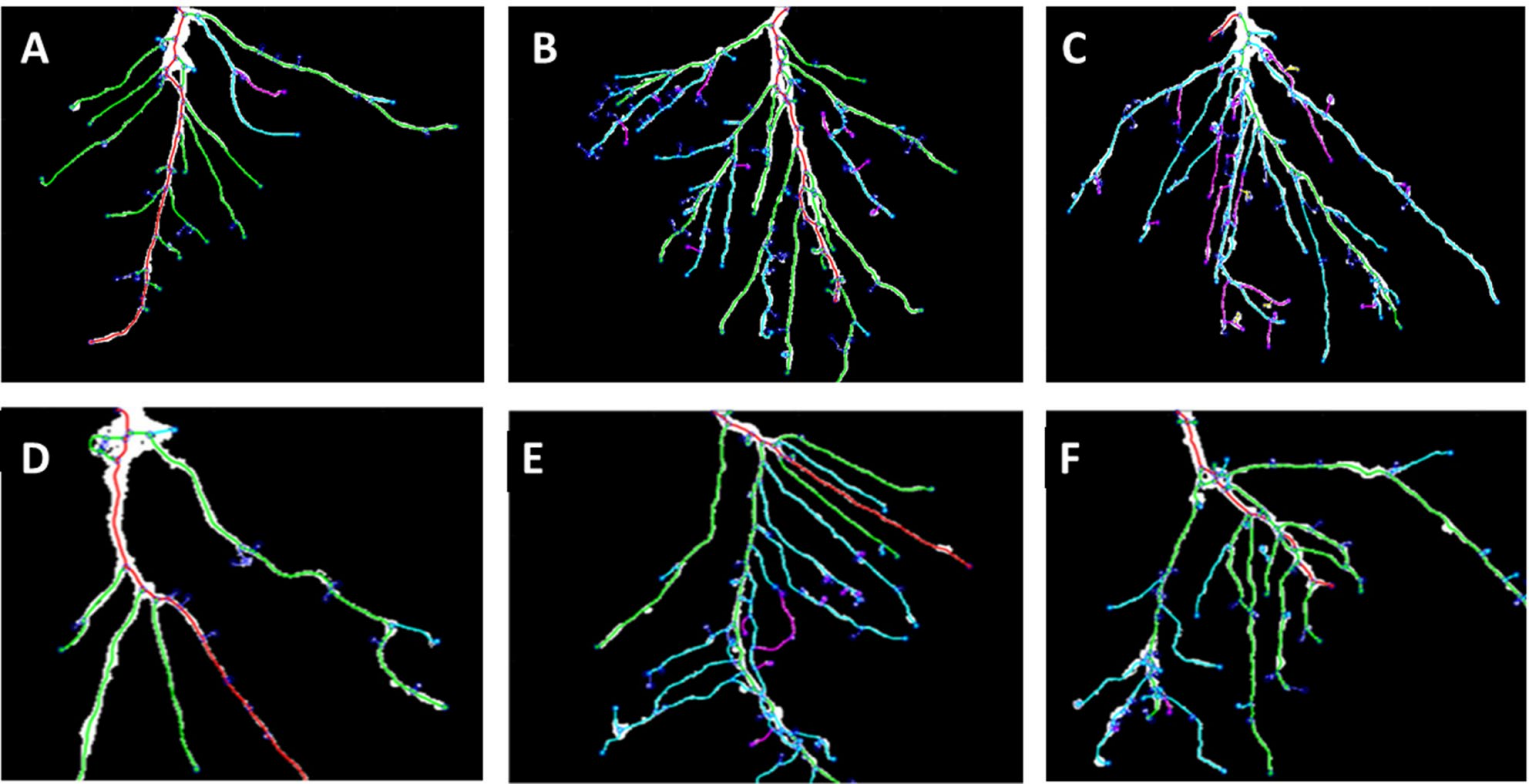
Root Architecture visualized by Root System Analyzer. Roots were collected after 8 days from the beginning of the treatment. The upper panels show the roots of unstressed plants after the application of water only (**A**), 1 mL L^−1^ VIVEMA TWIN (**B**) or 75 μM Gallic Acid (**C**). The lower panels show the roots of plants stressed with 100 mM NaCl and additionally treated with water only (**D**), 1 mL L^−1^ VIVEMA TWIN (**E**) or 75 μM Gallic Acid (**F**).

In order to understand the root development in the early growth phases both in the presence and in the absence of biostimulant or salt stress, imaging analyses were performed using Root System Analyzer software. Through the use of this software, we were able to convert a 2D picture in a black&white skeleton, in which roots of different orders are dissimilarly colored^41^. Consequently, we could visualize not only the root structure, but also compare the total root number of the different plant samples. Figure 2 shows a representative picture of roots from unstressed and salt-stressed plants after treatment with water, 1mLL^-1^ VIVEMA TWIN or 75 μM GA. We showed that, under standard conditions, plants treated with the biostimulant (Fig. 2B) have more developed root system in comparison to both control (Fig. 2A) and 75 μM GA treated (Fig. 2C) roots, due to the presence of a high number of lateral roots. When plants were stressed with 100 mM NaCl (Fig. 2D), the root architecture was strongly affected, with a consequent reduction in the size of the secondary roots. On the other hand, in salt stressed plants that were additionally treated with the biostimulant (Fig. 2E) or with 75 μM GA (Fig. 2F) alone, a restoration of the radical architecture was observed. In particular, this effect appeared to be more evident in plants treated with the biostimulant.

**Figure 3:**
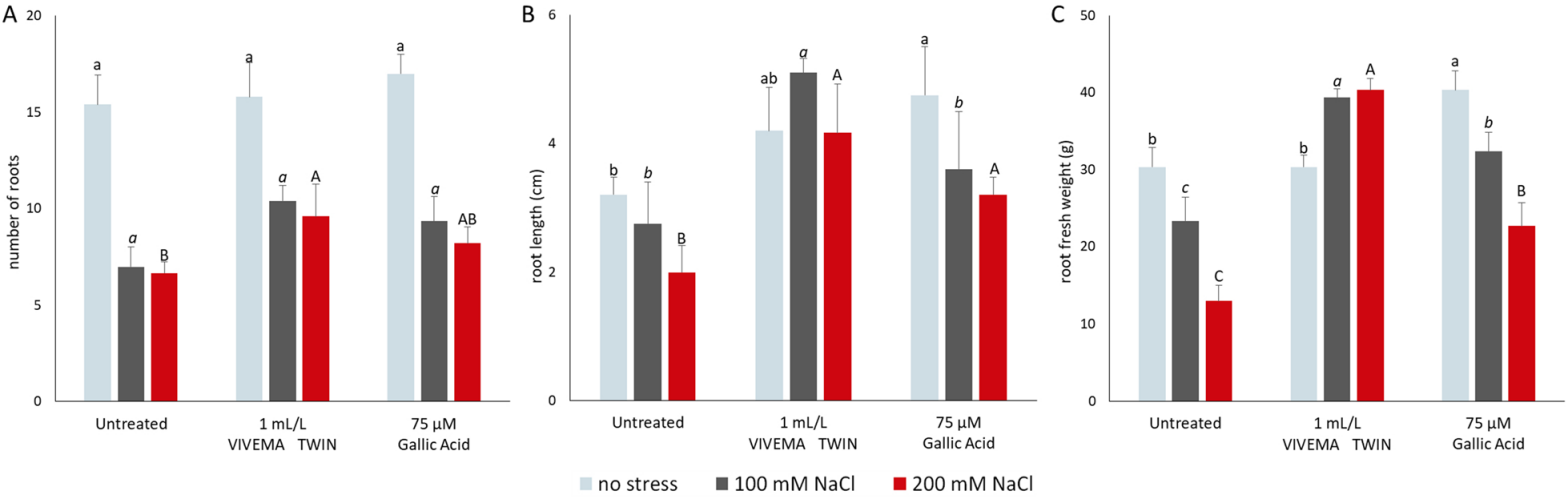
VIVEMA TWIN enhances the tomato root performance under salt stress. Total lateral root number (**A**), root length (**B**) and root fresh weight (**C**) of plant treated with 1 mL L^-1^ VIVEMA TWIN, 75 μM Gallic Acid, or water only. Roots were collected after 8 days from the beginning of the treatment. The biometric parameters were evaluated on both unstressed and 100 mM or 200 mM NaCl stressed plants. Bars represent the means ± SD of twenty biological replicates. Among the same series, statistical differences are indicated by different letters (ANOVA, Tukey–Kramer’s post-hoc test, *p* ≤ 0.05).

As shown on Fig. 3, plants grown in optimal conditions and treated with VIVEMA TWIN or 75 μM GA did not show a decrease in the lateral root number (Fig. 3A), length (Fig. 3B) or root fresh weight (Fig. 3C), suggesting the absence of negative effects of the biostimulant on the root architecture. Moreover, plants grown in absence of salt stress and treated with 75 μM GA showed an increased root length and fresh weight compared to the control and VIVEMA TWIN treated plants. Different effect on the root growth was observed in the presence of the stress. When salt stress was induced through the application of 100 mM or 200 mM NaCl, a strong and statistically significant decrease of all biometric parameters was recorded in plants not treated with the biostimulant or GA. The strongest stress effects were observed after the application of 200 mM NaCl. Comparable effects under similar experimental conditions were also previously observed and reported^42^. In this case, tomato plants subjected to different concentrations of NaCl (0, 50, 100 mM) showed a decrease of biometric parameters such as shoot fresh weight, plant height and number of leaves, in correlation with the higher salt concentration. Finally, when VIVEMA TWIN was applied on plants stressed with 100 mM NaCl, an increase of the root number (from 7.0 ± 1.1 to 10.4 ± 0.8), root length (from 2.8 ± 0.3 to 5.1 ± 0.6 cm) and root fresh weight (from 23.3 ± 3.1 to 39.3 ± 1.2 mg) was recorded. Similar effects were also displayed after the treatment with 75 μM GA, in which the number of roots increased up to 9.6 ± 1.7, their length to 4.2 ± 0.8 cm and their fresh weight to 40.3 ± 1.5 mg. Moreover, a comparable trend, although in lesser extent, was also observed in plants stressed with 200 mM NaCl and treated with the same dosage of biostimulant or Gallic Acid. Finally, the observed effects displayed after the application of the biostimulant, may not only be the result of a synergic action of the different chemical compounds present in the mixture, but it can also be originated from a protective antioxidant effects supplied by the application of VIVEMA TWIN. Moreover, our data show that this biostimulant can be used to improve the salt stress resilience in tomato, and perhaps in other crops as well.

### VIVEMA TWIN and gallic acid are able to modify the root architecture also in long-term salt stress treatment

**Figure 4:**
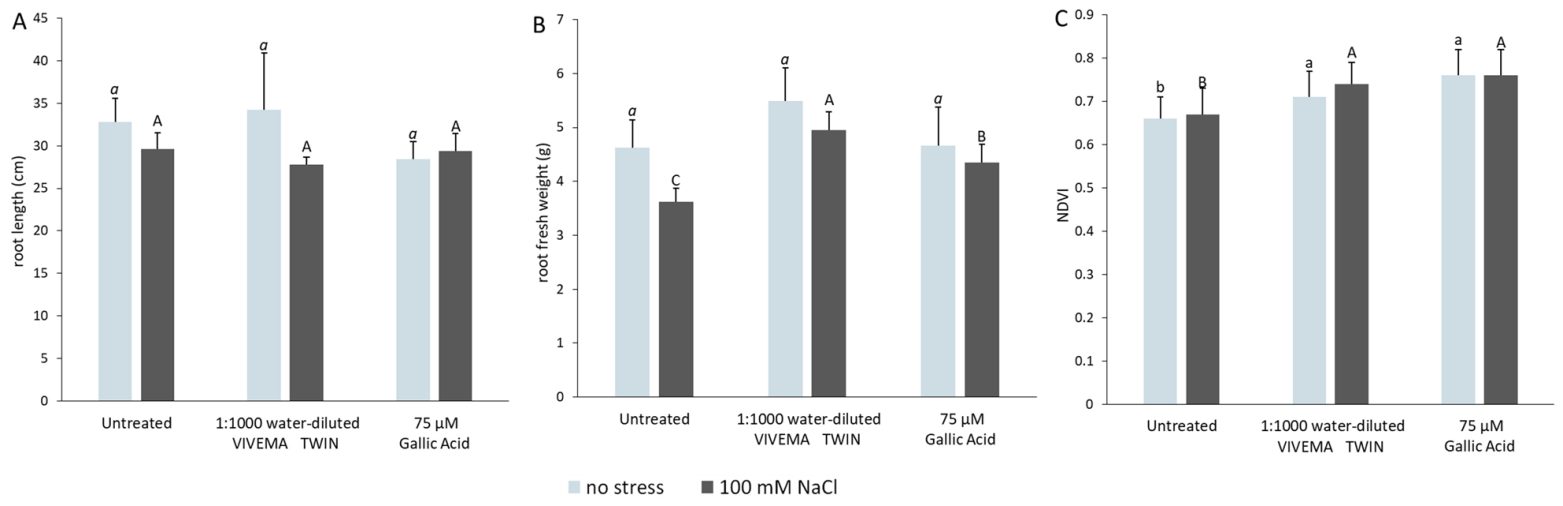
VIVEMA TWIN enhances the plant growth under salt stress. Root length (**A**), root fresh weight (**B**) and NDVI (**C**) of plants treated with 1 mL L^−1^ VIVEMA TWIN, 75 μM Gallic Acid, or water only. Roots were collected 4 weeks after the treatment, whereas NDVI index was measured before plant collection. The biometric parameters were evaluated on both unstressed and 100 mM NaCl stressed plants. Bars represent the means ± SD of twenty biological replicates. Among the same series, statistical differences are indicated by different letters (ANOVA, Tukey–Kramer’s post-hoc test, *p* ≤ 0.05).

The root development of tomato plants was also studied during the long-term stress treatment, by evaluating biometric parameters, such as root length, root fresh weight and NDVI index. NDVI is a unit designed to measure both red and near infrared reflectance on vegetation, two parameters useful to determine plant health. NDVI measurements can range from − 1 to 1, with higher values indicating better plant health^43^. The roots used for biometric data measurements were collected 24 h after the second (Supplementary Fig. 1) and the fourth (Fig. 4) biostimulant treatment. A parallel test, under the same experimental conditions, was also performed in order to compare the effects shown after the application of VIVEMA TWIN with those resulting from the application of 75 μM GA or water only. As showed in Supplementary Fig. 1, 24 h after the second plant treatment, 100 mM NaCl did not significantly affected root length and fresh weight, but strongly influenced NDVI. Indeed, in the second sampling time point, this value decreased from 0.72 ± 0.06 to 0.61 ± 008. On the other hand, the treatment with the biostimulant or with gallic acid was able to completely recover the NDVI index. Different effect was observed 24 h after the fourth treatment (Fig. 4). In this case, we did not observed any NDVI change between plants watered with 100 mM NaCl or with water only (Fig. 4C), although a strong reduction in the fresh weight was recorded upon salt treatment (Fig. 4B). The lack in the NDVI changes may be linked to a possible plant adaptation to a prolonged salt stress condition^44^. On the other hand, even thought NDVI was not negatively affected by 100 mM NaCl treatment, a significantly increased of NDVI was recorded after the application of VIVEMA TWIN or GA. In this case, the treatments led to even higher values in comparison to control plants grown in optimal conditions. Concerning root weight, after the application of the biostimulant or gallic acid, we observed a recovery of the negative effects caused by salt stress. Finally, no negative effects were observed on the root growth parameters after the treatment with the biostimulant or 75 μM GA when compared to unstressed plants.

### VIVEMA TWIN positively modulates the expression of genes involved in salt stress response, root growth and phosphate availability

In order to understand the molecular mechanism of action of VIVEMA TWIN, genome-wide expression (RNA-Seq analysis) was carried out on roots of 100 mM NaCl stressed-plants and treated with 1 mL L^−1^ VIVEMA TWIN. The roots of both conditions were collected 24 h after the last treatments. These data were compared to the gene expression of 100 mM NaCl stressed-plants which were treated with water instead of the biostimulant (control). Finally, Gene Ontology (GO) (Supplementary Fig. 2) and Kyoto Encyclopedia of Genes and Genomes (KEGG) (Supplementary Fig. 3) were conducted with the aim to identify the main biological functions and pathways of the differentially expressed key genes^45^.

The RNA-Seq analysis revealed the upregulation of 285 genes and the down-regulation of 171 genes, respectively upon the treatment with the biostimulant in the presence of salt stress. In Table 3 the significantly upregulated genes (FC > 1.7) are shown. Most of these genes are involved in abiotic stress response (63%), root growth (18.5%) and other metabolic functions (18.5%) based on GO analysis. The most significantly downregulated genes (1/FC > 1.7, FC < 0.6) are reported in Table 4.

**Table 3:** List of the most significantly upregulated genes (FC > 1.7;* p* < 0.05; n = 3) from RNA-Seq analysis on tomato roots.

Gene ID	Gene description	FC
**Abiotic stress response**		
Solyc12g056750.2	WRKY transcription factor 51	2.95
Solyc04g082550.2	Trehalose 6-phosphate phosphatase	2.76
Solyc02g077980.2	Late embryogenesis abundant protein (LEA) family protein	2.50
Solyc00g272810.1	Tyramine N-feruloyltransferase 4/11, putative	2.26
Solyc04g078900.3	ABA 8′-hydroxylase	2.25
Solyc05g024410.3	Na^+^/H^+^ exchanger 8	2.19
Solyc05g050220.3	TAF-3	2.16
Solyc04g009910.3	Phosphoenolpyruvate carboxylase kinase 1	2.09
Solyc10g084910.2	C_2_H_2_ zinc finger protein	2.08
Solyc04g071770.3	Ethylene-responsive transcription factor	2.07
Solyc12g010820.2	Late embryogenesis abundant protein-like	1.97
Solyc04g007470.3	Drought responsive Zinc finger protein	1.93
Solyc06g066540.1	Ethylene-responsive transcription factor TINY	1.93
Solyc02g081340.3	Glutathione S-transferase	1.92
Solyc07g040680.3	SolycHsfA9	1.92
Solyc06g076400.3	Protein phosphatase 2C	1.89
Solyc12g044390.2	Ethylene-responsive transcription factor	1.87
Solyc12g006050.2	Major facilitator superfamily protein	1.81
Solyc11g010930.2	HVA22-like protein	1.81
Solyc02g089190.2	R2R3MYB transcription factor 29	1.79
Solyc08g075320.3	Cytochrome P450 family ABA 8′-hydroxylase	1.78
Solyc06g084330.3	ERD (early-responsive to dehydration stress) family protein	1.77
Solyc10g081840.2	Nuclear transcription factor Y subunit	1.77
Solyc01g087590.3	Polyamine oxidase	1.76
Solyc12g006640.2	Lactoylglutathione lyase / glyoxalase I family protein	1.75
**Root growth**		
Solyc10g009580.3	Glycosyltransferase	2.48
Solyc12g042600.2	Glycosyltransferase	2.34
Solyc06g072870.2	Glycosyltransferase	2.01
Solyc07g054840.3	R2R3MYB transcription factor 41	2.34
Solyc10g084910.2	C_2_H_2_ zinc finger protein	2.08
Solyc04g076710.3	COBRA-like protein 11 precursor	1.90
Solyc12g006050.2	Major facilitator superfamily protein	1.81
Solyc08g005610.3	Xyloglucan endotransglucosylase-hydrolase 5	1.76

The fold change expression (FC) of genes from roots treated with 100 mM NaCl and 1 mL L^−1^ VIVEMA TWIN compared to untreated plants grown in the same conditions is shown.

**Table 4:** List of the most significantly downregulated genes (1/FC > 1.7, FC < 1.6) from the RNA-Seq analysis on tomato roots.

Gene ID	Gene description	FC
**Nutrient uptake**		
Solyc03g098010.3	Phosphate starvation inducible gene TPSI1	0.15
Solyc03g005530.1	Phosphate transporter	0.38
Solyc01g090890.3	SPX domain-containing protein	0.38
Solyc05g009640.3	bHLH transcription factor 037	0.47
Solyc08g060920.3	IDS4-like	0.50
Solyc02g091890.2	myb-like protein X	0.51
Solyc09g091910.2	Purple acid phosphatase	0.53
Solyc08g007800.3	SPX domain-containing family protein	0.54

Table reports the fold change (FC) expression of genes from roots treated with 100 mM NaCl and 1 mL L^−1^ VIVEMA TWIN compared to untreated plants.

#### Nutrient availability related genes

Some of these upregulated genes are suggested to be involved in nutrient availability, including the *Phosphate starvation inducible gene TPSI1* (FC = 0.145), *phosphate transporter* (FC = 0.377), *SPX domain-containing protein* (FC = 0.384), *bHLH transcription factor 037* (FC = 0.465), *IDS4-like* (FC = 0.504), *myb-like protein* (FC = 0.514) and *purple acid phosphatase* (FC = 0.534), which are involved in phosphate deficiency, a very important element for plant growth. Normally, these genes are induced in presence of phosphate starvation, while the decrease of their transcripts is observed when Pi-(inorganic phosphate) in starved tomato plants is resupplied. These data suggest that plants treated with VIVEMA TWIN, and grown under salt stress conditions, might have a better capacity to uptake phosphorous in comparison to untreated and stressed plants.

#### Abscisic acid (ABA) related genes

Several genes involved in ABA signaling were differentially regulated. These include *WRKY transcription factor* (FC = 2.95), *Trehalose 6-phosphate phosphatase* (*T6PP*) (FC = 2.76), *ABA 8′-hydroxylase* (FC = 2.25), *protein phosphatase 2C* (FC = 1.89) and *HVA22-like protein* (FC = 1.81). The upregulation of these genes is related to the increase of ABA activity, correlated to an increase of stress tolerance^46^. ABA, also called “stress hormone”, is fundamental in plant development and plays a key role both in the integration of stress signals and in the control of the stress response^47^. In particular, based on the plant status, WRKY transcription factors act as activators or repressors of ABA signaling, and are also involved in plant adaptation to salt stress^48,49^. Trehalose 6-phosphate phosphatase (T6PP) catalyzes the conversion of trehalose-6-P (T6P) to trehalose, a disaccharide involved in stress tolerance increase^50,51^. T6P, an highly soluble and low molecular weight compound, works as osmoprotectant by enhancing the resistance against salt stress^52^. Moreover, *T6PP* expression and trehalose content are increased in response to ABA and the synergistic action between the disaccharide and ABA also leads to a positive effect on root elongation in *Arabidopsis*^53^. ABA 8′-hydrolase, is a cytochrome P450 enzyme, involved in ABA catabolism, and importantly in maintaining the hormone balance^54^. The Protein phosphatase 2C was shown to play a key role in ABA signal transduction in *Arabidopsis* as well^55^, whereas HVA22-like protein in cereals is an ABA/stress induced protein, whose upregulation inhibits the formation of gibberellin GA-induced large vacuoles^56^. Our data suggest that in tomato similar ABA salt stress related signal transduction is activated for the survival of the plant due to the application of the biostimulant as compared to *Arabidopsis* and other species.

#### Late embryogenesis abundant (LEA) proteins

This group of genes is represented by two different *Late embryogenesis abundant protein (LEA) family proteins* (FC = 2.50 and FC = 1.97). Even if the mechanism of action of these proteins is not completely known in tomato, the upregulation of these genes in plants is strongly associated to the increment of stress tolerance, in particular in response to water limitation^57^. Drought stress presents many similarities with salt stress, since, in presence of salt, water is less available to the plants. The stress response involves the activation of common mechanisms, including the synthesis of LEA proteins^58^.

#### Other stress response related genes

Identified genes related to this category were *Na*^+^*/H*^+^ *exchanger 8 (NHE8)* (FC = 2.19), *C*_*2*_*H*_*2*_* zinc finger protein (C*_*2*_*H*_*2*_*ZnFP)* (FC = 2.08), *Drought responsive Zinc finger protein (DRZnFP)* (FC = 1.93) and *Glutathione-S-transferase (GST)* (FC = 1.92). *NHE8* is known to be involved in salt and drought stress in tomato plants, and in particular encodes for a plasma membrane antiporter, essential to maintain Na^+^/K^+^ homeostasis^59^. Its upregulation usually leads to the increase of salt stress tolerance in tomato plants^60^. *C*_*2*_*H*_*2*_*ZnFP* and *DRZnFP* are genes coding for transcription factors activated by stress conditions. With regard to glutathione (GST), this enzyme catalyzes the conjugation between reduced glutathione (GSH) and electrophilic substrates. In particular, these enzymes have been largely studied for their capacity to bind toxic exogenous compounds, thus protecting plants from multiple types of stresses^61,62^, including abiotic stress responses^62,63^, and their upregulation may suggest a protective antioxidant effect of VIVEMA TWIN application.

#### Root development related genes

A number of the genes upregulated by the biostimulant application were shown to be involved in the root developmental process. These are *R2R3MYB transcription factor 41* (FC = 2.34), *Major facilitator superfamily protein* (FC = 1.81) and *xyloglucan endotransglucosylase-hydrolase 5* (FC = 1.76). Different studies showed that these genes are involved in primary and lateral root formation, by acting on cellulose deposition (*xyloglucan endotransglucosylase-hydrolase 5*)^64^*,* auxin transport (*Major facilitator superfamily protein*)^65^ in *Arabidopsis* and in different other aspects like root hair development, root elongation and root architecture (*R2R3MYB transcription factor 41*)^66^. Finally, as reported above, also *T6PP* seems to have a role in root elongation.

#### Gene validation by qPCR analysis

RNA-Seq data were validated through qPCR analysis on selected genes significantly regulated by the application of the biostimulant (Supplementary Table 2). The qPCR analysis was performed on samples derived from VIVEMA TWIN-treated roots of plants grown under salt stress conditions (100 mM NaCl) and compared to the roots of stressed untreated plants. The roots were collected 24 h after the fourth treatment, at the same time point for which RNA-Seq analysis was carried out. Moreover, in order to compare the gene expression data related to the application of the biostimulant with those of the pure compound, qPCR was also performed on roots of stressed plants treated with 75 μM GA. Roots were collected at the same time point as previously described. The expression data are reported in Table 5.

**Table 5:** Comparison of RNA-Seq data with qPCR data.

Genes	1 mL L^−1^ VIVEMA TWIN		75 μM Gallic acid
	RNAseq	qPCR	qPCR
WRKY transcription factor 51	2.95	5.06*	4.23*
Trehalose 6-phosphate phosphatase	2.76	14*	0.81
Na^+^/H^+^ exchanger 8	2.19	1.8	1.19
C_2_H_2_ zinc finger protein	2.08	1.83	1.01
Glutathione S-transferase	1.92	27.03*	2.13*
Major facilitator superfamily protein	1.81	1.01	0.6
ERD (early-responsive to dehydration stress)	1.77	10.46*	1.99
Glycosyltransferase	2.48	21.59*	3.15*
Xyloglucan endotransglucosylase-hydrolase 5	1.76	1.55*	1.02
Phosphate starvation inducible gene TPSI1	0.15	0.81	1.5

In the table, the fold change (FC) value of each gene, obtained by comparing the gene expression of 100 mM NaCl stressed and untreated plants with the gene expression of plants treated with 1 mL L^−1^ VIVEMA TWIN or 75 μM Gallic Acid are shown. Statistical differences between treated plants and controls of each group in qPCR analysis are indicated by asterisks (Student’s *t*-test, *p* ≤ 0.05).

In general, the up/down regulation of selected genes observed in the RNA-Seq experiments was confirmed by qPCR analysis on the samples treated with the biostimulant. Differently, the gene expression of plants treated with 75 μM GA seems not to be significantly modulated by the treatment. Only *WRKY transcription factor 61*, *GST* and *GSyT* showed a significant (*p* ≤ 0.05) upregulation, similarly to plants treated with VIVEMA TWIN. Based on these results, the biostimulant seems to be effective on the expression of genes involved in stress response, root growth and nutrient uptake. Interestingly, the application of the pure GA showed a lower effect on gene expression, suggesting a possible action in synergy with other components present in VIVEMA TWIN mixture. The study of synergistic effect of compounds in a mixture is a current research topic in the biostimulant field. Indeed, not only the role of single components needs to be investigated, but also the effect resulting from their interactions in a complex mixture.

## Conclusions

**Figure 5:**
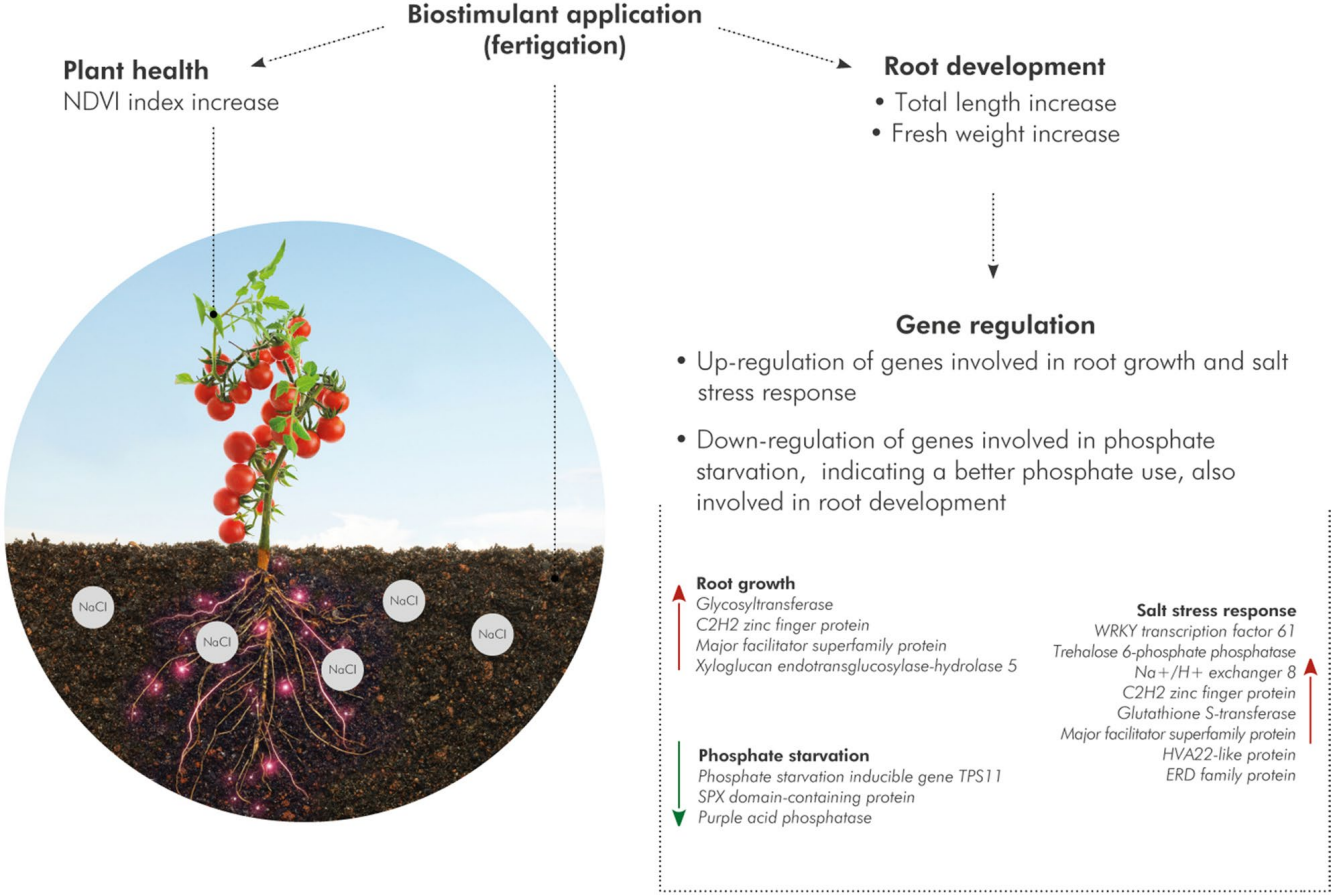
Schematic representation of the mode of action of VIVEMA TWIN. The biostimulant, applied by fertigation in the soil, leads to an increase of the NDVI index in the leaves and to a better root system development in tomato plants grown under salt stress conditions. RNA-Seq analysis on the treated roots revealed the upregulation of genes involved in root growth and salt stress response and the downregulation of genes linked to phosphate starvation, indicating that this biostimulant enhances the nutrient uptake of tomato roots.

In conclusion, our study presents novel insights on the modifications of root architecture in salt-stressed tomato plants after the treatment with the biostimulant VIVEMA TWIN. The development of root architecture with the concomitant modulation of genes involved in root growth, abiotic-stress responses and nutrient uptake, suggest that the biostimulant may have important role not only in plant nutrition but also in changing plant morphological traits for better plant salt stress survival and adaptation (Fig. 5).

Moreover, the comparison with GA treatments, showed how the synergistic activity of the biostimulant compounds led to more evident results on root development and plant resilience to salt stress than the application of a pure compound alone. Indeed, it has been shown that the effect of multiple bioactive molecules together is often higher than the sum of the effects of individual molecules^67^. Finally, it would be interesting to evaluate the effects of VIVEMA TWIN on other abiotic stresses and on different crops in order to verify its specificity and demonstrate its application potentially useful in multiple adverse conditions.

## Materials and methods

### Chemical characterization of VIVEMA TWIN

#### Spectrophotometric determination of bioactive compounds

The Total polyphenol content (TPC), Total Anthocyanin Content (TAnthC) and Total Flavan-3-ol content (TF3C) were determined spectrophotometrically using 1 mL L^-1^ of VIVEMA TWIN (Green Has Italia S.p.A., Canale, Italy) dissolved in water. Data are shown as mean of three biological replicates ± standard deviation (SD). TPC was determined by the reduction of phosphotungstic-phosphomolybdic acids (Folin-Ciocalteu’s reagent) to blue pigments in alkaline solution. The assays were carried out as previously described^24^, and the results were expressed as mg Gallic Acid Equivalents (GAE) per mL of biostimulant. TAnthC was determined via pH differential method, by reading the absorbance developed at 520 nm and 720 nm both at pH 1 and 4.5^68^. Data were expressed as mg of Cyanidine Equivalent (CyE) per mL of biostimulant. TF3C was determined by the formation of green-coloured complexes after the reaction of the flavan-3-ols with the BL-DMAC reagent. The assays were carried out as previously described^69^, and the results were expressed as PAC-A type equivalent per mL of biostimulant. Limit of detection (LOD) and Limit of Quantification (LOQ) were calculated for each assay using calibration curves of pure standards (Extrasynthese, France), ranging between 1–20 μg mL^−1^. LOD and LOQ were calculated as previously reported^69,70^.

#### HPLC-ESI-FTMS identification and quantification

VIVEMA TWIN was analyzed by using an Orbitrap Fourier Transformed Mass Spectrometer (FTMS; Thermo Fisher) hybrid system. The biostimulant was firstly 1:1000 (v/v) diluted in water, and then mixed 1:2 (v/v) with 50% (v/v) methanol containing 1% (v/v) formic acid. Samples were vortexed (XH-D lab Vortex Mixer, Scientific Instrument) and sonicated (Ultrasonic Bath P120H, Elma) for 10 min, and then centrifuged (Sorvall Primo, Thermo Fisher) for 15 min at 10.000 g. The obtained supernatant was transferred to a new microcentrifuge tube, and used for further analysis. The chromatographic separation was carried out using a LUNA 3 μm C18(2), 150 × 2.00 mm column (Phenomenex, USA) and with a linear gradient from 5 to 35% B in 45 min, using a flow rate of 0.20 mL min^−1^, as previously described^71^. The FTMS was set at a mass resolution of 60,000 HWHM, and with a mass range of m/z 140–2000. Electrospray ionization (ESI) in negative mode was employed for the ionization of compounds. Identification and quantification was based on retention times (RT) and accurate masses (MW) compared to the pure standards (5–20 µg mL^−1^) of ellagic acid, gallic acid and tannic acid. Data analysis was performed following the methods previously described^71,72^.

### Evaluation of the antioxidant properties of VIVEMA TWIN

#### Ferric reducing antioxidant power (FRAP)

The reducing activity of VIVEMA TWIN was evaluated by FRAP assay measuring the reduction of the Fe^3+^–TPTZ complex to the ferrous form^24^. Briefly, the FRAP reactive, prepared by mixing 0.3 M acetate buffer (pH 3.6), 10 mM 2,4,6-Tripyridyl-S-triazine (TPTZ), and 20 mM FeCl_3_ in 8:1:1 (v/v/v) ratio, was incubated at 37 °C for 30 min with a proper sample dilution and the absorbance was measured at 595 nm. All measurements were repeated three times. Gallic Acid was used as a reference compound, and data were expressed as mmol of GAE per mL of biostimulant.

#### Radical scavenging activity (ABTS and DPPH)

The radical scavenging property of VIVEMA TWIN was evaluated by ABTS (2,2′-azino-bis(3-ethylbenzothiazoline-6-sulfonic acid) and DPPH (2,2-diphenyl-1-picrylhydrazyl) assay. ABTS radical cation decolorization assay was performed as previously described^73^. The assays are based on monitoring the colorization decay of the radical forms (ABTS^·+^ or DPPH) respectively at 515 or 735 nm. For both assays, samples were analyzed at five different dilutions, within the linearity range of the assay. Gallic acid was used as a reference compound, and the reducing activity was expressed as mmol GAE per mL of biostimulant. All measurements were repeated three times.

### Plant material and treatment with biostimulant

**Figure 6:**
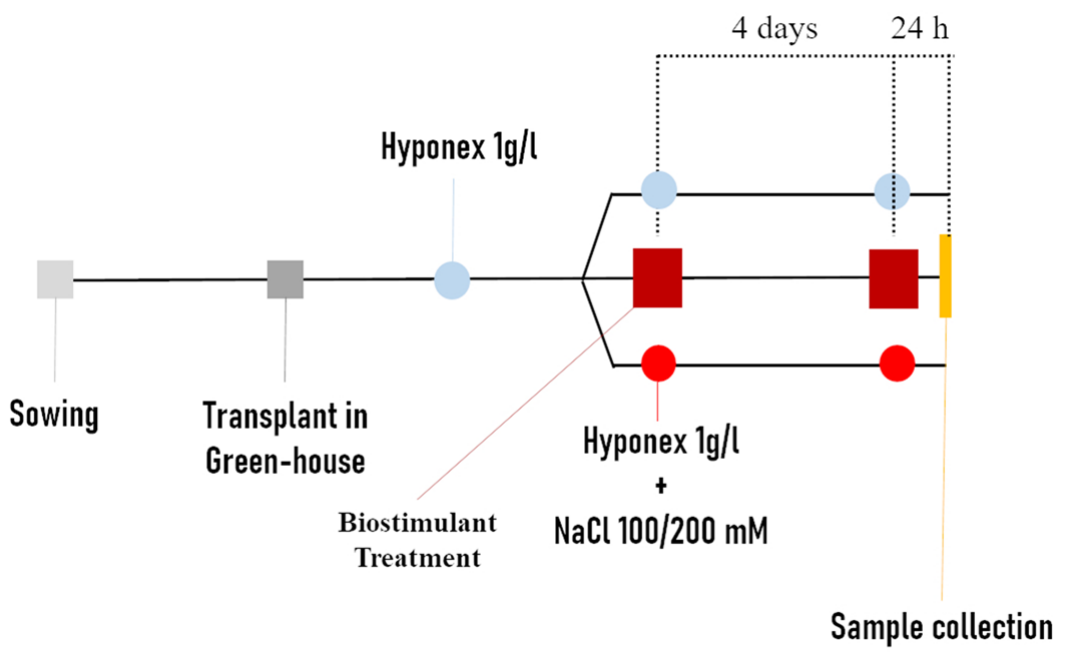
Schematization of short-term test. Seeds were sown in plates, then seedlings transferred to the green house and watered with Hyponex as nutrient solution. After the first true leaf appearance, the plants were treated or not with salt stress. After 4 days, the treatment was repeated following the same experimental conditions. Salt stressed plants were watered with 100 or 200 mM NaCl solution at the same time of the water/biostimulant treatment.

**Figure 7:**
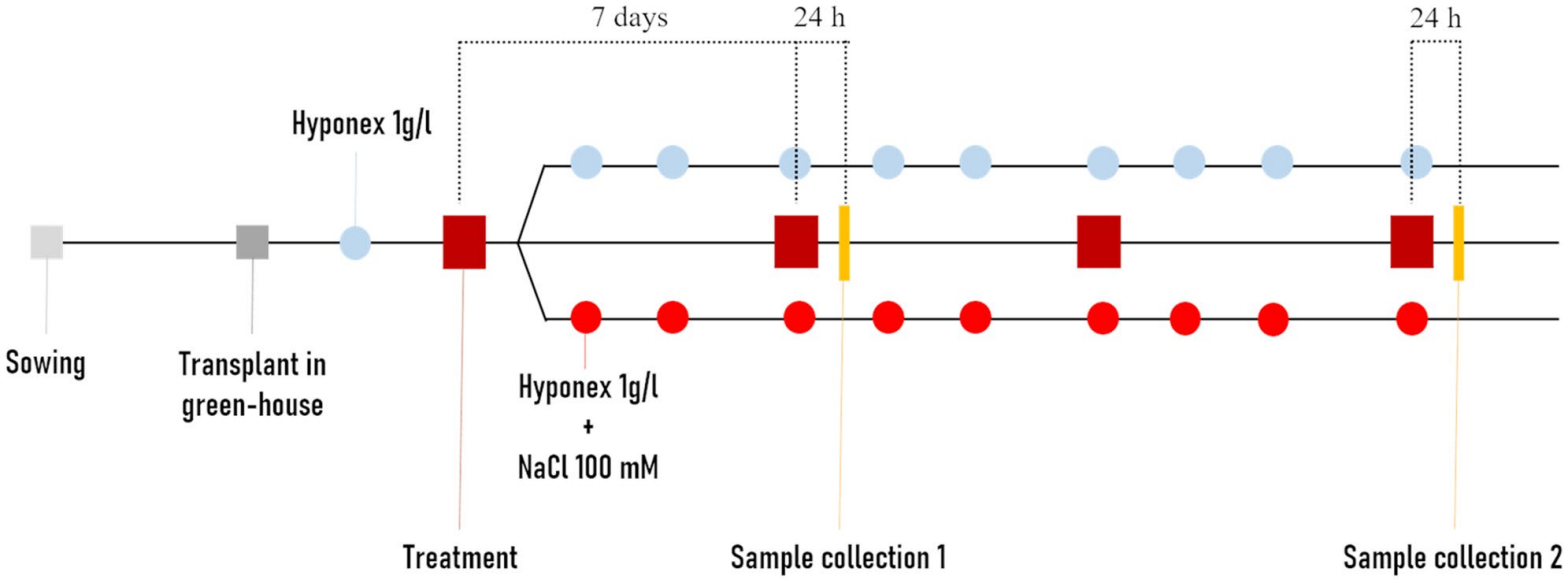
Schematization of Long-term test. Seeds were sown in plate, then seedlings transferred to the green house and watered with Hyponex as nutrient solution. After the first true leaf appearance, the plants were treated, under optimal or salt stress conditions. After 7, 14 and 21 days, the treatment was repeated following the same experimental conditions. Salt stressed plants were watered with 100 mM NaCl solution three times a week.

Tomato (*S. lycopersicum* L. Heinz 1706) seeds were sown in plate on a wet filter paper. Plates were incubated in a growth chamber (25 °C, 16/8 h light/dark, PPFD 100 μmol m^−2^ s^−1^) for 7 days. Seedlings were then transferred in the greenhouse in pots containing 100% sand. The pots were watered three times a week with 1 g L^−1^ nutrition solution (Hyponex, Japan). After the first leaf emergence (BBCH 11), plants were treated by application of water (untreated control), 1 mL L^−1^ VIVEMA TWIN (Green Has Italia S.p.A., Canale (CN), Piedmont, Italy) (treated samples) or 75 μM GA. The different treatments were also performed under standard or salt stress conditions. For each growth condition (unstressed/untreated, unstressed/treated, stressed/untreated, stressed/treated), twenty plants were used, randomly distributed, considering each plant as a biological replicate using a fully randomized experimental design. For “short-term” test (Fig. 6), plants were treated every 4 days, starting 2 days after transplantation in the greenhouse. In this case, salt stress was induced by watering plants with 100 mM or 200 mM NaCl solutions in two parallel experiments. Because of the shortness of the experiment, the saline solution was given at the same time of each treatment. Roots were collected 24 h after the second treatment. For “long-term” tests (Fig. 7), plants were treated once per week, for 4 weeks and the salt stress was induced after the first treatment (priming treatment) by watering the plants three times a week with 100 mM NaCl added to the nutrition solution. For the stressed plants, no further irrigations were carried out, other than those with salt. Leaves and roots were collected 24 h after the second treatment and 24 h after the fourth one.

### Evaluation of morphological parameters

#### Short-term test

After washing and drying the roots, length of the primary root was manually measured, and the fresh weight was determined using an analytical scale. Moreover, the picture of each root was processed with Root System Analyzer. This is an automated approach employed to measure root architectural parameters from two-dimensional root images^41,74^. The data are obtained in the form of original photos, which are consequently converted into a black&white skeleton. In the skeleton each root order is indicated with a different color. The total number of roots, measured according to the procedure described above, was also taken into account.

#### Long-term test

Normalized Difference Vegetation Index (NDVI) of both untreated and treated plants was monitored on leaves before the plant material collection, using Pigment Analyzer PA110 (Control in Applied Physiology, Germany). Plants were then collected, and root length was manually measured, and the root fresh weight was obtained using an analytical scale. Dry weight was determined by washing and drying the roots in an oven at 70 °C for 48 h.

### Genome expression analysis

#### Total RNA isolation

Total RNA was isolated from powdered roots using TRIzol reagent (Thermo Fisher Scientific, MA, USA) and following the manufacturer’s instructions. RNA quality was evaluated by 1% (w/v) agarose gel electrophoresis, whereas its purity was measured by NanoPhotometer (IMPLEN, USA).

#### RNA-Seq analysis

RNA-Seq analysis was performed by Novogene (Hong Kong, China) both on roots untreated or treated with VIVEMA TWIN, grown under salt stress conditions (100 mM NaCl). Before proceeding with library construction the total RNA integrity was checked by using Nano 6000 Assay Kit and Agilent 2100 Bioanalyzer 2100 (Agilent Technologies, USA) and the concentration measured using Qubit RNA Assay Kit in Qubit 2.0 Fluorometer (Life Technologies, USA).

Three µg of total RNA was used as input material for the generation of the sequence libraries. Transcriptome assembly, coding potential analysis, expression analysis, GO enrichment and KEGG pathway analysis, were done using Novogene standardized protocols as already shown before in other similar studies^62,75–77^. Raw counts produced by HTSeq-count were normalized based on the DESeq2 normalization method^78^. DESeq2 linear models in R environment were implemented in order to identify statistically significant differentially expressed genes (DEGs) between treated samples and controls. Genes with an adjusted (Benjamini–Hochberg correction for multiple hypothesis testing) *p* value < 0.05 were considered as differentially expressed. Moreover, Log2FC cut-off was set to 1.7. The list of all DEGs obtained from this experimentation is reported in Supplementary Table 3 Finally, RNA-Seq data were submitted to SRA (Sequence Read Archive) database with the accession number PRJNA645803.

#### cDNA synthesis and qPCR analysis

RNA-Seq data were validated through qPCR analysis carried out on the most significantly regulated genes after the application of VIVEMA TWIN (*WRKY transcription factor 61*, *Trehalose 6-phosphate phosphatase*, *Na*^+^*/H*^+^ *exchanger 8*, *C*_*2*_*H*_*2*_* zinc finger protein*, *Glutathione S-transferase*, *Major facilitator superfamily protein*, *ERD, Glycosyltransferase*, *Xyloglucan-endotransglucosylase-hydrolase 5*, *TPSI1*). The same genes were also analyzed in GA-treated roots in order to evaluate gene expression variations between the biostimulant and the pure compound. Briefly, half a µg of RNA was reverse transcribed by using the Maxima H Minus First Strand cDNA Synthesis kit (Thermo Fisher Scientific, USA), following the manufacturer’s instructions. For each qPCR reaction 0.3 μM primers, 4.1 μL of nuclease-free H_2_O and 5 μL of SYBR-Green I (Maxima SYBR Green/ROX qPCR Master Mix 2X, Thermo Fisher Scientific, USA) were added. The qPCR reactions were performed using a QuantStudio 1 Real-Time PCR System (Thermo Fisher Scientific, USA). All the conditions used for the different gene amplification are reported in Supplementary Table 1. For each treatment, three biological replicates (obtained by combining the twenty biological replicates in three pools) and three technical replicates were analyzed. To normalize the data, four different reference genes (*Tubulin*, *Ubiquitin*, *Elongation Factor 1* and a *catalytic subunit of Protein Phosphatase 2A*) were also analyzed. Among these, a reference was selected using the Normfinder software^79^; the most stable gene selected was the *Elongation Factor 1.* The primers of both reference and selected target genes, were designed using Primer3 software^80^ and are reported in Supplementary Table 2. The relative gene expression levels were calculated by using the Pfaffl method^62,81^, meanwhile the variations in gene expression were calculated as relative quantification of the target genes in relation to the reference gene, *Elongation Factor 1* as shown before^24,62^.

### Statistical analysis

Data, if not differently specified, were analyzed through one-way analysis of the variance (ANOVA) using “Systat Version 10” (Systat Software, San Jose, CA). Tukey–Kramer’s post-hoc test (*p* ≤ 0.05) was used to analyze the means. For each experiment at least twenty biological replicates were used. When necessary, L1-norm exclusion test was performed with the aim to remove outliers from the data set. In this case, data of at least sixteen biological replicates were used for further statistical analysis.

## Supplementary information

Supplementary Information.
