# Supplementary material for: The application of a biostimulant based on tannins affects root architecture and improves tolerance to salinity in tomato plants
Source: Sci Rep. 2021 Jan 11;11:354. doi: 10.1038/s41598-020-79770-5 (PMC7801735; doi:10.1038/s41598-020-79770-5)
Supplement: Supplementary file 1 — Supplementary Information. [file 41598_2020_79770_MOESM1_ESM.pdf]

The application of a biostimulant based on tannins affects root architecture and improves tolerance to salinity in tomato plants

Cristina Campobenedetto[1,2,3b], Giuseppe Mannino[1], Jules Beekwilder[3a], Valeria Contartese[2], Rumyana Karlova[3b*], Cinzia M. Bertea[1*]

[1] Plant Physiology Unit, Department of Life Sciences and Systems Biology, University of Torino, Turin, Italy; cristina.campobenedetto@unito.it (CC); giuseppe.mannino@unito.it (GM); cinzia.bertea@unito.it (CMB)
[2] Green Has Italia S.p.A, Canale (CN), Italy; c.campobenedetto@greenhasitalia.it (CC); v.contartese@greenhasitalia.it (VC)
[3a] Wageningen University and Research Centre, Bioscience, Wageningen, The Netherlands; jules.beekwilder@wur.nl (JB)
[3b] Laboratory of Plant Physiology, Plant Sciences Group, Wageningen University and Research, 6708PB, 9 Wageningen, The Netherlands; rumyana.karlova@wur.nl (R.K.)
* Correspondence: rumyana.karlova@wur.nl and cinzia.bertea@unito.it; Tel.: +39-011-670-6361

Supplementary Files

Supplementary Table 1: qPCR conditions used for the amplification of both target and reference genes.

Phase	Time	Temperature	
Initial denaturation	10 min	95°C	
Denaturation	15 sec	95°C	
Annealing	20 sec	57°C	45X
Extension	30 sec	72°C	
	60 sec	95°C	
Melting curve	30 sec	55°C	
	30 sec	95°C	

Supplementary Table 2: Nucleotide primer sequences of reference and target genes used for the qPCR analysis.

Accession Number	ID (SolGenomics)	Genes			Nucleotide Sequences
XM_004252513.4	Solyc12g056750	*WRKY transcription factor 61*	*WRKYTF61*	**F**	AGTGTTCAATTCGAGGATGCA
				**R**	AGCTTTCATGATTGTGTTTGCCT
XM_015217355.1	Solyc04g082550	*Trehalose 6-phosphate phosphatase*	*T6PP*	**F**	TGGAACAAAGGTCATGCATTGG
				**R**	TGGTGTCTTTTGGAGCAGCA
XM_006358777.2	Solyc05g024410	Na^+/H^+ *exchanger 8*	*NHE8*	**F**	TGATTTGCAAGGATGTGCTCC
				**R**	CTAGCGGCTTTCCCAATCGA
AK329746.1	Solyc10g084910	C_2H_2 *zinc finger protein*	*C2H2ZnFP*	**F**	TTAGAAAACAAGGGCATGGAGC
				**R**	GAAGTTCAAATCGTGGATCTGTG
XM_015210287.2	Solyc02g081340	*Glutathione S-transferase*	*GSF*	**F**	AAGCCACCTCCCTAGCCTTA
				**R**	TGGGCCGGGTAACATCTCTA
XM_004251487.4	Solyc12g006050	*Major facilitator superfamily protein*	*MFSP*	**F**	AACAACGAAGGGAAAACGCG
				**R**	TCGAGTTGTCCAACGTAGGC
XM_015223407.2	Solyc06g084330	*Early-responsive to dehydration stress*	*ERD*	**F**	TGAGCACCAAAGAGGCTTCC
				**R**	ACTGTATCCTTCACCACCGC
XM_015233991.2	Solyc10g009580	*Glycosyltransferase*	*GST*	**F**	ATGTGTCGGTTGGTGGATTT
				**R**	GTCTGCAAAGAGAGGCCAAG
XM_015228135.2	Solyc08g005610	*Xyloglucan endotransglucosylase-hydrolase 5*	*XETGH*	**F**	TCCCCGGTACACTCTTCAAC
				**R**	ATGAATGACCCGAGCAAATC
XM_004235321.4	Solyc03g098010	*Phosphate starvation inducible gene*	*TPSI1*	**F**	GATCATTGCCTTGAGCACAT
				**R**	TTCAACCCTTTCACGTCTCC

Supplementary Figure 1: Root lenght (**A**), root fresh weight (**B**) and NDVI (**C**) of plants treated with 1 mL L^{-1} VIVEMA TWIN, 75 µM Gallic Acid, or water. Roots were collected 2 weeks after the treatment, whereas NDVI index was measured before plant collection. The biometric parameters were evaluated on both unstressed and 100 mM NaCl stressed plants. Bars represent the means ± SD of twenty biological replicates. Among the same series, statistical differences are indicated by different letters (ANOVA, Tukey-Kramer's post-hoc test, $p \leq 0.05$).

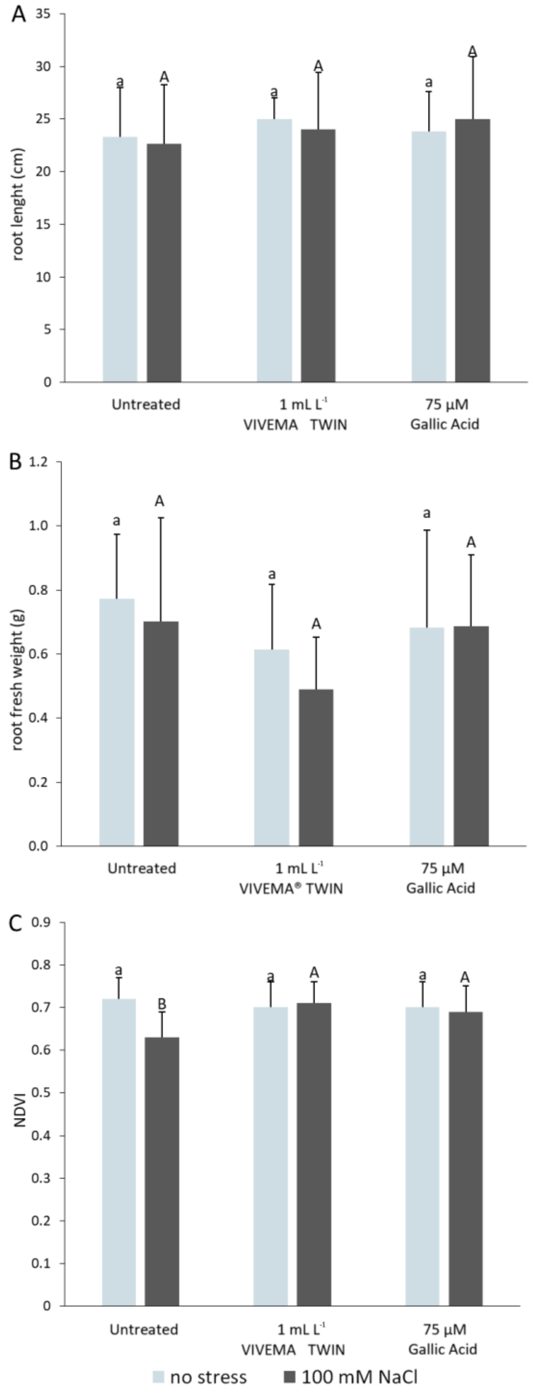

Supplementary Figure 2: Gene Ontology (GO) enrichment bar charts of differentially expressed genes (DEGs) of tomato plants that were treated with 1 mL L^{-1} VIVEMA TWIN and 100 mM salt stress or only with 100 mM salt stress (control). Panel A shows the top 30 upregulated enriched GO terms, meanwhile Panel B the top 30 downregulated GO terms. In both panels, the colors represent different GO types, and in particular those involved in biological process (green color) or in cellular component and molecular function (orange color).

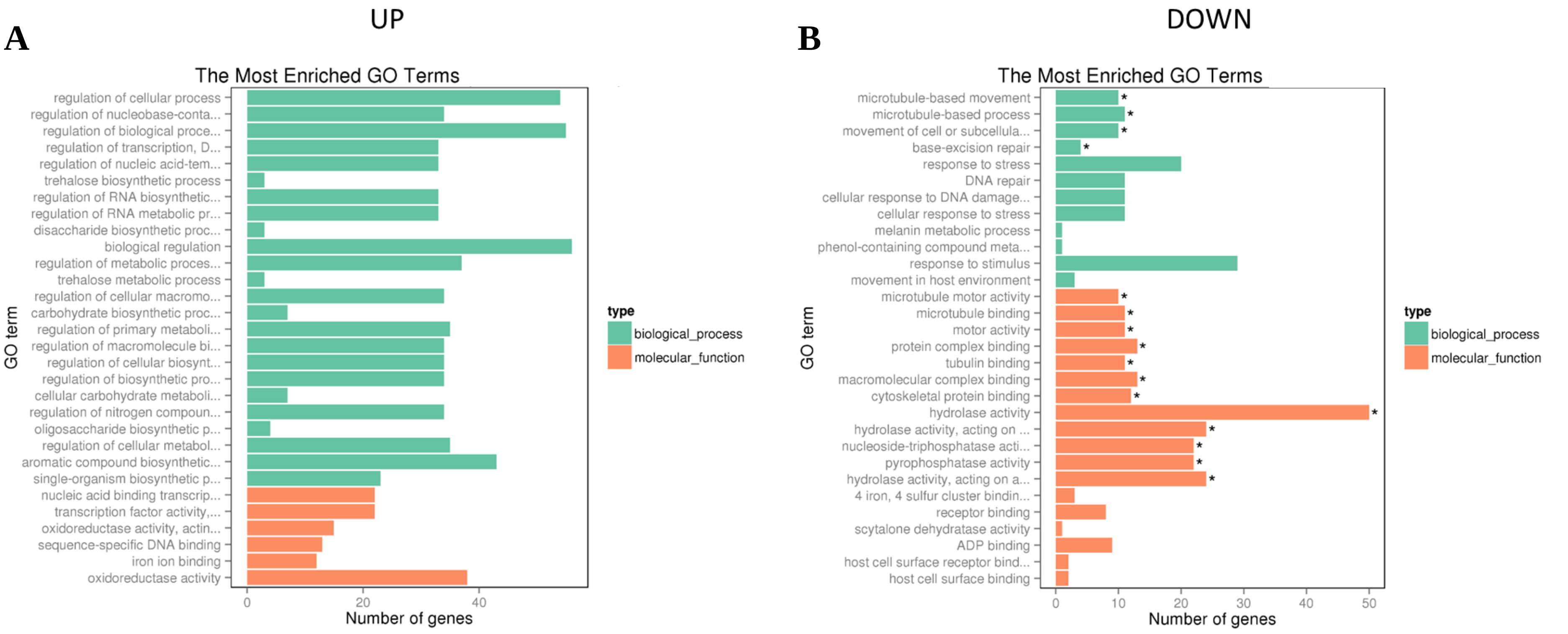

Supplementary Figure 3: Kyoto Encyclopedia of Genes and Genomes (KEGG) enrichment scatter plots of plants treated with 100 mM NaCl stress and 1 mL L^{-1} VIVEMA TWIN or only with salt stress (control). Panel A displays the 20 most significant enriched pathways for upregulated genes, meanwhile Panel B the most significant 20 enriched pathways for downregulated genes. The differentially expressed genes were grouped into gene pathways using the pathway enrichment analysis with the KEGG database and KOBAS software. In both panels, X-axis represents the name of the pathway and the Y-axis represents the Rich Factor. The size stands for the number of difference genes and the colour stands for different Q-values. The size of the circle is proportional to the number of enriched genes.

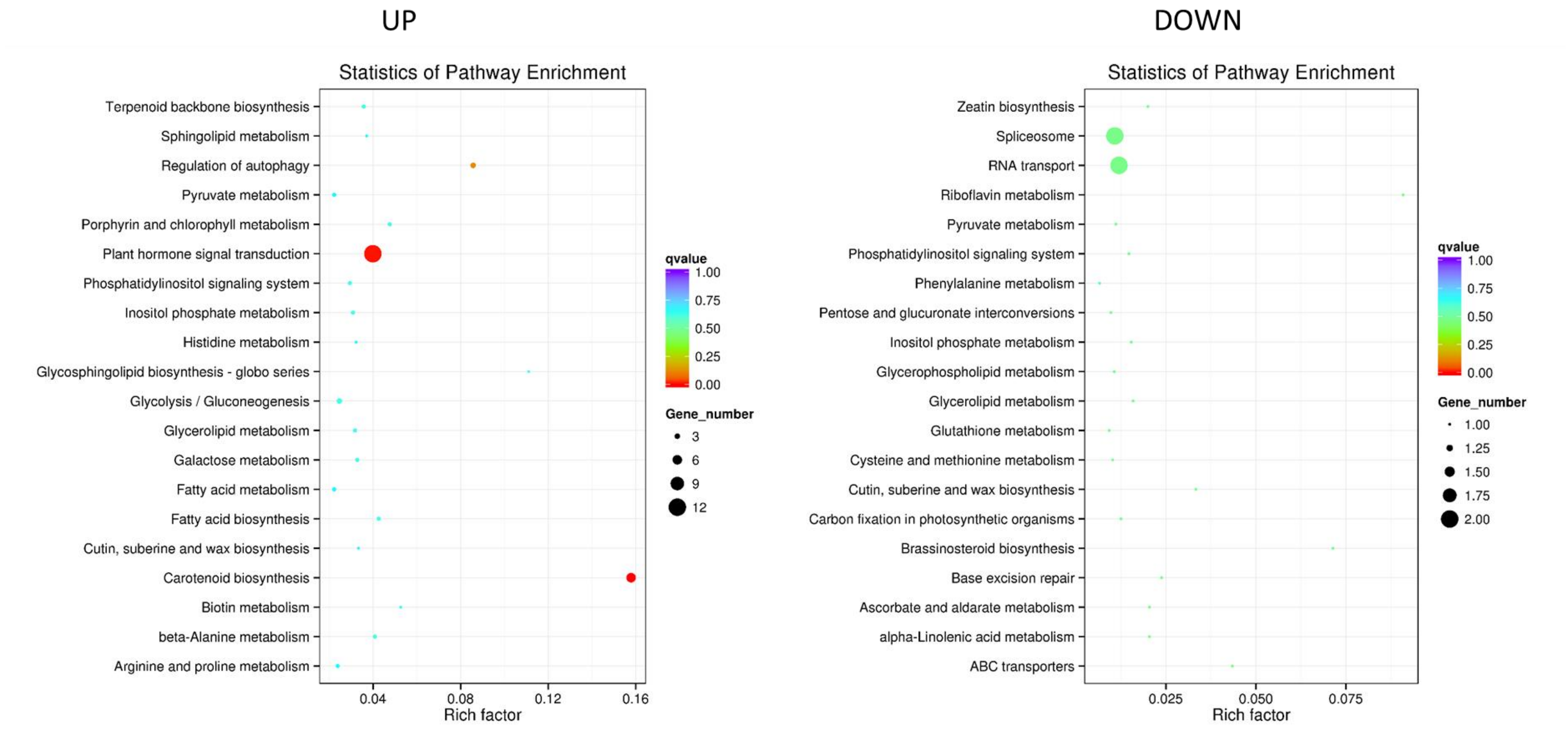

Supplementary Table 3: List of all Differentially Expressed Genes (DEGs) found in plant roots treated with 100 mM NaCl and 1 mL L^{-1} VIVEMA TWIN versus roots treated with 100 mM NaCl only. For each Gene, the ID (gene identification code), FC (fold change), log_2FC (log_2 fold change), pval (*p* value), padj (*p* value adjusted) are reported.

Gene_id	log_2FC	FC	pval	padj
Solyc05g015890.3	2.86	7.24	0.00	0.03
Solyc10g076670.2	1.70	3.26	0.00	0.04
Solyc12g096930.2	1.65	3.13	0.00	0.00
Solyc06g051560.3	1.57	2.97	0.00	0.00
Solyc12g056750.2	1.56	2.95	0.00	0.00
Solyc11g011340.2	1.52	2.86	0.00	0.01
Solyc04g082550.2	1.46	2.76	0.00	0.00
Solyc02g077980.2	1.32	2.50	0.00	0.00
Solyc03g007340.3	1.32	2.50	0.00	0.02
Solyc05g016710.2	1.32	2.49	0.00	0.05
Solyc10g009580.3	1.31	2.48	0.00	0.00
Solyc01g057170.3	1.30	2.47	0.00	0.00
Solyc06g050800.3	1.30	2.46	0.00	0.02
Novel00272	1.25	2.39	0.00	0.02
Solyc12g042600.2	1.23	2.34	0.00	0.01
Solyc07g054840.3	1.22	2.34	0.00	0.01
Solyc00g272810.1	1.18	2.26	0.00	0.00
Solyc04g078900.3	1.17	2.25	0.00	0.03
Solyc02g079290.3	1.16	2.23	0.00	0.02
Solyc10g055460.2	1.14	2.21	0.00	0.00
Solyc05g024410.3	1.13	2.19	0.00	0.01
Solyc05g050220.3	1.11	2.16	0.00	0.00
Solyc05g055460.1	1.10	2.15	0.00	0.00
Solyc04g009910.3	1.06	2.09	0.00	0.00
Solyc10g084910.2	1.06	2.08	0.00	0.02
Solyc04g071770.3	1.05	2.07	0.00	0.00
Solyc03g025720.3	1.03	2.04	0.00	0.01
Solyc06g072870.2	1.01	2.01	0.00	0.00
Solyc12g010820.2	0.98	1.97	0.00	0.02
Novel00183	0.96	1.95	0.00	0.00
Solyc02g083000.1	0.96	1.94	0.00	0.03
Solyc04g007470.3	0.95	1.93	0.00	0.01
Solyc06g066540.1	0.95	1.93	0.00	0.00
Solyc02g081340.3	0.94	1.92	0.00	0.01
Solyc07g040680.3	0.94	1.92	0.00	0.00
Solyc04g076710.3	0.92	1.90	0.00	0.00
Solyc06g076400.3	0.92	1.89	0.00	0.00
Solyc12g044390.2	0.90	1.87	0.00	0.02
Solyc01g109120.3	0.90	1.87	0.00	0.00
Solyc01g006720.3	0.90	1.86	0.00	0.00
Solyc11g066880.2	0.88	1.84	0.00	0.00
Solyc09g097960.3	0.88	1.84	0.00	0.00
Solyc12g006050.2	0.86	1.81	0.00	0.02

Solyc11g010930.2	0.85	1.81	0.00	0.01
Solyc08g013900.3	0.85	1.80	0.00	0.00
Solyc04g005380.3	0.84	1.80	0.00	0.00
Solyc02g089190.2	0.84	1.79	0.00	0.00
Solyc08g075320.3	0.84	1.78	0.00	0.00
Solyc03g111100.1	0.83	1.77	0.00	0.00
Solyc06g084330.3	0.83	1.77	0.00	0.00
Solyc10g081840.2	0.82	1.77	0.00	0.00
Solyc08g005610.3	0.82	1.76	0.00	0.00
Solyc01g087590.3	0.81	1.76	0.00	0.00
Solyc06g072710.3	0.81	1.76	0.00	0.00
Solyc12g006640.2	0.81	1.75	0.00	0.04
Solyc04g015200.3	0.80	1.75	0.00	0.00
Solyc05g054890.3	0.78	1.72	0.00	0.05
Solyc07g062520.3	0.78	1.72	0.00	0.02
Solyc10g080730.2	0.78	1.71	0.00	0.00
Solyc11g005930.2	0.77	1.70	0.00	0.05
Solyc04g071140.3	0.77	1.70	0.00	0.00
Solyc01g096320.3	0.76	1.70	0.00	0.00
Solyc05g009320.3	0.76	1.70	0.00	0.01
Solyc03g118450.1	0.76	1.70	0.00	0.01
Solyc11g011970.2	0.76	1.69	0.00	0.00
Solyc06g051550.3	0.74	1.67	0.00	0.00
Solyc07g006130.2	0.74	1.67	0.00	0.00
Solyc04g081900.3	0.74	1.66	0.00	0.01
Solyc04g076950.3	0.73	1.66	0.00	0.00
Solyc09g009150.1	0.73	1.66	0.00	0.00
Solyc01g096280.2	0.71	1.64	0.00	0.00
Solyc01g080580.3	0.71	1.64	0.00	0.00
Solyc03g120020.3	0.71	1.63	0.00	0.01
Solyc04g072890.3	0.70	1.63	0.00	0.04
Novel00572	0.70	1.63	0.00	0.00
Solyc03g115850.3	0.70	1.62	0.00	0.01
Solyc04g074950.3	0.70	1.62	0.00	0.01
Solyc12g013620.2	0.70	1.62	0.00	0.01
Solyc12g088230.2	0.70	1.62	0.00	0.00
Solyc03g096670.3	0.69	1.62	0.00	0.00
Solyc07g065060.2	0.68	1.61	0.00	0.04
Solyc08g075280.3	0.68	1.60	0.00	0.01
Solyc01g107240.3	0.68	1.60	0.00	0.02
Solyc03g116530.3	0.67	1.59	0.00	0.03
Solyc02g078210.3	0.67	1.59	0.00	0.03
Solyc06g048750.3	0.67	1.59	0.00	0.03
Solyc12g095810.2	0.64	1.56	0.00	0.00
Solyc01g107170.2	0.64	1.56	0.00	0.04
Solyc07g063420.3	0.64	1.56	0.00	0.03
Solyc06g010060.1	0.64	1.56	0.00	0.00
Solyc10g005460.3	0.64	1.55	0.00	0.00
Solyc11g010170.2	0.63	1.55	0.00	0.01

Solyc03g007370.3	0.63	1.55	0.00	0.00
Solyc02g093890.1	0.63	1.55	0.00	0.01
Solyc03g116280.3	0.63	1.55	0.00	0.00
Solyc07g064900.3	0.63	1.54	0.00	0.00
Solyc10g083670.2	0.63	1.54	0.00	0.00
Solyc03g118130.3	0.63	1.54	0.00	0.04
Solyc02g090890.3	0.62	1.54	0.00	0.00
Solyc08g065500.2	0.62	1.54	0.00	0.00
Solyc03g120640.3	0.62	1.54	0.00	0.00
Solyc10g007070.3	0.61	1.53	0.00	0.00
Solyc06g071060.2	0.61	1.53	0.00	0.00
Solyc04g072895.1	0.61	1.53	0.00	0.00
Solyc11g016940.2	0.61	1.53	0.00	0.02
Solyc03g116710.3	0.61	1.52	0.00	0.01
Solyc07g006850.2	0.61	1.52	0.00	0.00
Solyc05g008390.3	0.61	1.52	0.00	0.01
Solyc08g007080.3	0.61	1.52	0.00	0.00
Solyc12g007070.2	0.60	1.52	0.00	0.00
Solyc01g058260.3	0.60	1.51	0.00	0.00
Solyc02g088910.3	0.60	1.51	0.00	0.00
Solyc09g065020.3	0.59	1.51	0.00	0.05
Solyc07g065810.2	0.59	1.51	0.00	0.01
Solyc03g121880.3	0.59	1.51	0.00	0.00
Solyc04g081290.3	0.59	1.50	0.00	0.00
Solyc02g071090.3	0.59	1.50	0.00	0.03
Solyc04g054930.3	0.59	1.50	0.00	0.00
Solyc02g076920.3	0.58	1.50	0.00	0.00
Solyc02g081330.3	0.58	1.49	0.00	0.00
Solyc04g056310.3	0.58	1.49	0.00	0.00
Solyc09g090790.3	0.57	1.49	0.00	0.00
Solyc09g005660.3	0.57	1.48	0.00	0.00
Solyc03g116270.3	0.57	1.48	0.00	0.00
Solyc04g076820.1	0.56	1.47	0.00	0.00
Solyc04g053130.3	0.56	1.47	0.00	0.05
Solyc11g008030.2	0.55	1.47	0.00	0.03
Solyc10g079620.2	0.55	1.47	0.00	0.00
Solyc04g077980.1	0.54	1.46	0.00	0.00
Solyc02g081170.3	0.54	1.45	0.00	0.00
Solyc04g008210.2	0.54	1.45	0.00	0.00
Solyc01g111570.3	0.53	1.45	0.00	0.00
Solyc01g096880.3	0.53	1.45	0.00	0.00
Solyc05g052980.3	0.53	1.44	0.00	0.01
Solyc02g070670.3	0.53	1.44	0.00	0.00
Solyc12g089220.2	0.53	1.44	0.00	0.00
Solyc01g009935.1	0.52	1.44	0.00	0.01
Novel00122	0.52	1.43	0.00	0.02
Solyc12g005880.2	0.52	1.43	0.00	0.02
Solyc11g010120.2	0.52	1.43	0.00	0.00
Solyc01g106253.1	0.52	1.43	0.00	0.04

Solyc11g013083.1	0.51	1.43	0.00	0.01
Solyc07g043590.3	0.51	1.43	0.00	0.00
Solyc02g090310.1	0.51	1.43	0.00	0.00
Solyc02g086860.3	0.51	1.42	0.00	0.00
Solyc02g085610.3	0.51	1.42	0.00	0.00
Solyc03g121870.3	0.51	1.42	0.00	0.03
Solyc11g018690.2	0.51	1.42	0.00	0.00
Solyc10g080980.2	0.51	1.42	0.00	0.01
Solyc05g012210.3	0.50	1.42	0.00	0.00
Solyc04g078730.2	0.50	1.42	0.00	0.01
Solyc03g095310.3	0.50	1.42	0.00	0.03
Solyc04g016060.1	0.50	1.42	0.00	0.00
Solyc02g079280.3	0.50	1.41	0.00	0.02
Solyc03g093270.3	0.50	1.41	0.00	0.03
Solyc04g081020.3	0.50	1.41	0.00	0.01
Solyc04g078360.2	0.50	1.41	0.00	0.00
Solyc01g079865.1	0.50	1.41	0.00	0.02
Solyc07g065250.3	0.49	1.41	0.00	0.00
Solyc02g094180.3	0.49	1.41	0.00	0.03
Solyc09g011370.2	0.49	1.41	0.00	0.00
Solyc08g065610.3	0.49	1.41	0.00	0.00
Solyc08g067310.1	0.49	1.41	0.00	0.02
Solyc07g040990.3	0.49	1.40	0.00	0.00
Solyc10g083460.1	0.49	1.40	0.00	0.04
Solyc11g045610.2	0.49	1.40	0.00	0.01
Solyc06g005070.2	0.48	1.40	0.00	0.04
Solyc12g013810.2	0.48	1.40	0.00	0.01
Solyc06g066600.3	0.48	1.40	0.00	0.02
Solyc12g019740.2	0.47	1.39	0.00	0.02
Solyc08g023440.3	0.47	1.39	0.00	0.00
Solyc04g078850.3	0.47	1.39	0.00	0.01
Solyc06g007120.3	0.47	1.39	0.00	0.00
Solyc01g103990.3	0.47	1.39	0.00	0.03
Solyc11g005560.2	0.47	1.39	0.00	0.00
Solyc01g108840.3	0.47	1.39	0.00	0.01
Solyc04g079300.3	0.47	1.39	0.00	0.00
Solyc06g005470.3	0.47	1.39	0.00	0.04
Solyc02g085670.3	0.47	1.38	0.00	0.02
Solyc01g095460.3	0.46	1.38	0.00	0.00
Solyc01g079650.3	0.46	1.38	0.00	0.01
Solyc02g089115.1	0.46	1.37	0.00	0.01
Solyc06g053960.3	0.46	1.37	0.00	0.01
Solyc07g016170.3	0.45	1.37	0.00	0.00
Solyc03g113270.3	0.45	1.37	0.00	0.00
Solyc05g006980.3	0.45	1.37	0.00	0.00
Solyc06g050300.2	0.45	1.37	0.00	0.02
Solyc02g089120.3	0.45	1.36	0.00	0.02
Solyc08g077440.3	0.45	1.36	0.00	0.02
Solyc06g068450.2	0.45	1.36	0.00	0.00

Solyc04g054370.1	0.44	1.36	0.00	0.03
Solyc01g006780.3	0.44	1.36	0.00	0.00
Solyc06g062960.2	0.44	1.36	0.00	0.04
Solyc12g006930.2	0.44	1.36	0.00	0.04
Solyc12g008660.1	0.44	1.36	0.00	0.00
Solyc03g044910.1	0.44	1.36	0.00	0.00
Solyc09g092460.3	0.44	1.36	0.00	0.00
Solyc02g091930.3	0.44	1.36	0.00	0.00
Solyc03g115980.1	0.44	1.35	0.00	0.04
Solyc07g044990.3	0.44	1.35	0.00	0.01
Solyc12g038160.2	0.44	1.35	0.00	0.00
Solyc05g024160.3	0.43	1.35	0.00	0.00
Solyc11g012000.2	0.43	1.35	0.00	0.00
Solyc02g069470.3	0.43	1.35	0.00	0.01
Solyc01g096420.3	0.43	1.35	0.00	0.00
Solyc05g007220.3	0.43	1.35	0.00	0.04
Solyc02g085700.2	0.43	1.35	0.00	0.00
Solyc09g075560.1	0.43	1.35	0.00	0.00
Solyc03g117070.1	0.43	1.35	0.00	0.01
Solyc12g089240.2	0.43	1.35	0.00	0.00
Solyc01g006790.2	0.43	1.35	0.00	0.01
Solyc01g079160.3	0.43	1.34	0.00	0.05
Solyc12g056590.2	0.43	1.34	0.00	0.05
Solyc08g082870.3	0.43	1.34	0.00	0.02
Solyc01g010660.3	0.42	1.34	0.00	0.03
Solyc05g014260.3	0.42	1.34	0.00	0.00
Solyc06g068720.3	0.42	1.34	0.00	0.02
Solyc06g074940.3	0.42	1.34	0.00	0.02
Solyc04g006960.3	0.42	1.34	0.00	0.00
Solyc09g075020.3	0.41	1.33	0.00	0.00
Solyc01g108087.1	0.41	1.33	0.00	0.01
Solyc02g078830.3	0.41	1.33	0.00	0.02
Solyc04g063380.3	0.41	1.33	0.00	0.01
Solyc12g015770.2	0.41	1.33	0.00	0.01
Solyc04g054320.3	0.41	1.33	0.00	0.00
Solyc01g097470.3	0.41	1.32	0.00	0.03
Solyc09g074660.3	0.41	1.32	0.00	0.04
Solyc01g105270.3	0.40	1.32	0.00	0.05
Solyc07g054270.3	0.40	1.32	0.00	0.05
Solyc02g071860.3	0.40	1.32	0.00	0.01
Solyc02g091430.3	0.40	1.32	0.00	0.00
Solyc07g024000.3	0.40	1.32	0.00	0.02
Solyc03g007960.3	0.40	1.32	0.00	0.01
Solyc12g005850.2	0.40	1.32	0.00	0.04
Solyc05g015610.3	0.40	1.32	0.00	0.00
Solyc05g013660.3	0.39	1.31	0.00	0.03
Solyc02g083310.3	0.39	1.31	0.00	0.01
Solyc05g021390.3	0.39	1.31	0.00	0.03
Solyc02g084610.1	0.39	1.31	0.00	0.04

Solyc04g025940.3	0.39	1.31	0.00	0.01
Solyc11g013180.2	0.39	1.31	0.00	0.00
Solyc03g083615.1	0.39	1.31	0.00	0.02
Solyc03g118425.1	0.39	1.31	0.00	0.05
Solyc08g081030.1	0.39	1.31	0.00	0.01
Solyc01g111260.2	0.39	1.31	0.00	0.01
Solyc02g081030.3	0.38	1.30	0.00	0.01
Solyc08g061920.2	0.38	1.30	0.00	0.04
Solyc01g087240.3	0.38	1.30	0.00	0.02
Solyc12g056650.2	0.38	1.30	0.00	0.01
Solyc07g007120.3	0.38	1.30	0.00	0.01
Solyc01g079870.3	0.37	1.30	0.00	0.01
Solyc00g009110.3	0.37	1.30	0.00	0.03
Solyc01g099980.3	0.37	1.30	0.00	0.01
Solyc04g079190.3	0.37	1.29	0.00	0.03
Solyc03g121960.3	0.37	1.29	0.00	0.03
Solyc01g067280.3	0.37	1.29	0.00	0.02
Solyc11g069580.2	0.37	1.29	0.00	0.04
Solyc11g072660.2	0.36	1.29	0.00	0.02
Solyc12g089160.2	0.36	1.29	0.00	0.04
Solyc11g073300.2	0.36	1.28	0.00	0.03
Solyc10g007030.2	0.36	1.28	0.00	0.02
Solyc03g119610.1	0.36	1.28	0.00	0.02
Solyc04g074580.1	0.36	1.28	0.00	0.01
Solyc04g012160.3	0.35	1.28	0.00	0.03
Solyc03g007270.3	0.35	1.28	0.00	0.02
Solyc06g050130.3	0.35	1.28	0.00	0.03
Solyc05g015390.3	0.35	1.28	0.00	0.01
Solyc12g005270.2	0.35	1.27	0.00	0.01
Solyc08g077780.3	0.35	1.27	0.00	0.01
Solyc12g006230.2	0.35	1.27	0.00	0.03
Solyc06g076350.3	0.35	1.27	0.00	0.03
Solyc07g052670.3	0.34	1.27	0.00	0.01
Solyc10g076480.2	0.34	1.27	0.00	0.03
Solyc03g097630.3	0.34	1.27	0.00	0.05
Solyc11g013750.2	0.34	1.26	0.00	0.03
Solyc01g106780.3	0.33	1.26	0.00	0.05
Solyc04g080790.3	0.33	1.26	0.00	0.03
Solyc08g074390.3	0.33	1.26	0.00	0.05
Solyc01g095620.3	0.33	1.26	0.00	0.02
Solyc03g111640.3	0.33	1.25	0.00	0.03
Solyc09g008790.3	0.32	1.25	0.00	0.04
Solyc03g111310.3	0.32	1.25	0.00	0.03
Solyc11g072540.2	0.32	1.25	0.00	0.04
Solyc01g086970.3	0.31	1.24	0.00	0.05
Solyc06g082530.2	0.30	1.23	0.00	0.04
Solyc03g123620.3	-0.31	0.81	0.00	0.03
Solyc12g045030.2	-0.33	0.80	0.00	0.03
Solyc12g005940.2	-0.33	0.79	0.00	0.04

Solyc04g008680.3	-0.35	0.79	0.00	0.02
Solyc08g007620.2	-0.36	0.78	0.00	0.03
Solyc09g091670.3	-0.36	0.78	0.00	0.01
Solyc03g093260.3	-0.36	0.78	0.00	0.05
Solyc01g010230.2	-0.37	0.78	0.00	0.02
Solyc03g097490.3	-0.37	0.78	0.00	0.04
Solyc01g103650.3	-0.37	0.77	0.00	0.03
Solyc02g078670.3	-0.37	0.77	0.00	0.01
Solyc02g087980.3	-0.37	0.77	0.00	0.04
Solyc04g008040.3	-0.37	0.77	0.00	0.02
Solyc07g065880.3	-0.37	0.77	0.00	0.03
Solyc05g054480.3	-0.38	0.77	0.00	0.02
Solyc09g010850.3	-0.38	0.77	0.00	0.03
Solyc04g080600.3	-0.38	0.77	0.00	0.04
Solyc10g007290.3	-0.40	0.76	0.00	0.00
Solyc08g008370.3	-0.40	0.76	0.00	0.01
Solyc03g083240.3	-0.41	0.75	0.00	0.02
Solyc06g075580.3	-0.41	0.75	0.00	0.02
Solyc01g073950.3	-0.42	0.75	0.00	0.02
Solyc06g076040.3	-0.42	0.75	0.00	0.00
Solyc03g033500.3	-0.42	0.75	0.00	0.03
Solyc02g067660.3	-0.42	0.75	0.00	0.01
Solyc11g007770.2	-0.42	0.75	0.00	0.02
Solyc06g059870.1	-0.42	0.75	0.00	0.01
Solyc01g014840.3	-0.43	0.74	0.00	0.02
Solyc11g007900.2	-0.43	0.74	0.00	0.02
Solyc11g012410.2	-0.43	0.74	0.00	0.00
Solyc01g081540.3	-0.43	0.74	0.00	0.02
Solyc11g072570.2	-0.44	0.74	0.00	0.03
Solyc11g007580.2	-0.44	0.74	0.00	0.02
Solyc12g005790.2	-0.44	0.74	0.00	0.00
Solyc03g058380.3	-0.44	0.74	0.00	0.00
Solyc07g032230.3	-0.44	0.74	0.00	0.00
Solyc01g058010.3	-0.44	0.74	0.00	0.01
Solyc03g119220.3	-0.44	0.73	0.00	0.02
Solyc09g009080.3	-0.45	0.73	0.00	0.00
Solyc10g008400.2	-0.45	0.73	0.00	0.02
Solyc01g111980.3	-0.45	0.73	0.00	0.00
Solyc03g083010.3	-0.45	0.73	0.00	0.01
Solyc06g009783.1	-0.46	0.73	0.00	0.03
Solyc03g045050.3	-0.46	0.73	0.00	0.00
Solyc03g097190.3	-0.46	0.73	0.00	0.00
Solyc05g005620.3	-0.47	0.72	0.00	0.00
Solyc02g077710.1	-0.47	0.72	0.00	0.02
Solyc10g005000.3	-0.47	0.72	0.00	0.00
Solyc12g005590.2	-0.47	0.72	0.00	0.00
Solyc03g058350.3	-0.47	0.72	0.00	0.00
Solyc10g008790.3	-0.47	0.72	0.00	0.04
Solyc06g005170.3	-0.47	0.72	0.00	0.00

Solyc06g060680.2	-0.47	0.72	0.00	0.00
Solyc12g098840.2	-0.47	0.72	0.00	0.00
Solyc05g007110.2	-0.47	0.72	0.00	0.04
Solyc03g119940.3	-0.47	0.72	0.00	0.02
Solyc03g005620.3	-0.47	0.72	0.00	0.04
Solyc05g053770.3	-0.48	0.72	0.00	0.01
Solyc12g011290.2	-0.48	0.72	0.00	0.01
Solyc05g011970.3	-0.48	0.72	0.00	0.02
Solyc06g008400.2	-0.48	0.71	0.00	0.01
Solyc12g100270.2	-0.49	0.71	0.00	0.00
Solyc05g008780.3	-0.49	0.71	0.00	0.00
Solyc02g085035.1	-0.49	0.71	0.00	0.00
Solyc07g006320.3	-0.50	0.71	0.00	0.01
Solyc04g007070.3	-0.50	0.71	0.00	0.01
Solyc11g006640.2	-0.50	0.71	0.00	0.03
Solyc11g010920.2	-0.50	0.71	0.00	0.01
Solyc06g067870.3	-0.50	0.70	0.00	0.00
Solyc08g007970.3	-0.51	0.70	0.00	0.00
Solyc06g062330.2	-0.51	0.70	0.00	0.01
Solyc06g008790.3	-0.51	0.70	0.00	0.01
Solyc04g071660.3	-0.51	0.70	0.00	0.00
Solyc04g072450.3	-0.51	0.70	0.00	0.00
Solyc03g119250.3	-0.52	0.70	0.00	0.04
Solyc07g018070.3	-0.52	0.70	0.00	0.00
Solyc09g091960.3	-0.52	0.70	0.00	0.02
Solyc06g072730.3	-0.52	0.70	0.00	0.02
Solyc03g115920.3	-0.52	0.70	0.00	0.04
Solyc05g044490.2	-0.53	0.69	0.00	0.03
Solyc08g080780.3	-0.53	0.69	0.00	0.00
Solyc11g069070.2	-0.53	0.69	0.00	0.01
Solyc04g081470.3	-0.53	0.69	0.00	0.01
Solyc11g071730.2	-0.53	0.69	0.00	0.04
Solyc07g056170.3	-0.54	0.69	0.00	0.00
Solyc11g071300.2	-0.54	0.69	0.00	0.02
Solyc02g062890.2	-0.54	0.69	0.00	0.02
Solyc04g071360.3	-0.54	0.69	0.00	0.01
Solyc01g057310.3	-0.54	0.69	0.00	0.05
Solyc11g008080.2	-0.55	0.69	0.00	0.02
Solyc09g005860.3	-0.55	0.68	0.00	0.00
Solyc04g015220.3	-0.55	0.68	0.00	0.04
Solyc03g032210.3	-0.55	0.68	0.00	0.00
Solyc05g006730.3	-0.55	0.68	0.00	0.00
Solyc10g083630.2	-0.56	0.68	0.00	0.00
Solyc04g025650.3	-0.56	0.68	0.00	0.00
Solyc06g082030.3	-0.56	0.68	0.00	0.03
Solyc03g119600.1	-0.56	0.68	0.00	0.00
Solyc10g018200.2	-0.56	0.68	0.00	0.03
Solyc11g044340.2	-0.58	0.67	0.00	0.00
Solyc04g015210.3	-0.58	0.67	0.00	0.00

Solyc07g065600.3	-0.58	0.67	0.00	0.00
Solyc02g093720.3	-0.59	0.67	0.00	0.01
Solyc12g014590.2	-0.59	0.67	0.00	0.00
Solyc02g021260.2	-0.59	0.67	0.00	0.03
Solyc12g014030.1	-0.60	0.66	0.00	0.03
Solyc12g009480.2	-0.60	0.66	0.00	0.03
Solyc05g010680.2	-0.60	0.66	0.00	0.01
Solyc07g053640.1	-0.61	0.65	0.00	0.01
Solyc04g007030.3	-0.62	0.65	0.00	0.01
Solyc08g082010.3	-0.62	0.65	0.00	0.02
Solyc07g041290.2	-0.63	0.65	0.00	0.00
Solyc07g009030.3	-0.63	0.65	0.00	0.00
Solyc02g071560.3	-0.64	0.64	0.00	0.02
Solyc12g013790.2	-0.65	0.64	0.00	0.00
Solyc07g065490.3	-0.65	0.64	0.00	0.00
Solyc12g014040.1	-0.66	0.64	0.00	0.00
Solyc05g014270.3	-0.67	0.63	0.00	0.01
Solyc02g069750.2	-0.67	0.63	0.00	0.00
Solyc01g108670.3	-0.70	0.62	0.00	0.00
Solyc05g055220.1	-0.71	0.61	0.00	0.04
Solyc01g102310.3	-0.73	0.60	0.00	0.05
Solyc04g005170.1	-0.73	0.60	0.00	0.04
Solyc12g036390.2	-0.74	0.60	0.00	0.00
Solyc12g005480.2	-0.74	0.60	0.00	0.02
Solyc07g049560.3	-0.75	0.59	0.00	0.00
Solyc01g057320.2	-0.76	0.59	0.00	0.01
Solyc01g100270.3	-0.76	0.59	0.00	0.00
Solyc06g072660.3	-0.77	0.58	0.00	0.01
Solyc06g016750.3	-0.78	0.58	0.00	0.03
Solyc06g069300.1	-0.79	0.58	0.00	0.01
Solyc04g082120.3	-0.79	0.58	0.00	0.04
Solyc01g090210.3	-0.83	0.56	0.00	0.00
Solyc06g059990.3	-0.84	0.56	0.00	0.00
Solyc05g007470.3	-0.84	0.56	0.00	0.00
Solyc03g123390.3	-0.84	0.56	0.00	0.00
Solyc09g010550.3	-0.86	0.55	0.00	0.00
Solyc08g082070.3	-0.87	0.55	0.00	0.00
Solyc03g093340.3	-0.87	0.55	0.00	0.04
Solyc08g007800.3	-0.88	0.54	0.00	0.01
Solyc07g008980.3	-0.89	0.54	0.00	0.01
Solyc04g082220.2	-0.89	0.54	0.00	0.02
Solyc09g091910.2	-0.91	0.53	0.00	0.00
Solyc07g006290.2	-0.91	0.53	0.00	0.01
Solyc10g085100.2	-0.95	0.52	0.00	0.00
Solyc02g091890.2	-0.96	0.51	0.00	0.00
Solyc10g007280.3	-0.96	0.51	0.00	0.00
Solyc11g010620.2	-0.97	0.51	0.00	0.00
Solyc07g054220.1	-0.99	0.50	0.00	0.00
Solyc08g060920.3	-0.99	0.50	0.00	0.02

Solyc03g025830.3	-0.99	0.50	0.00	0.00
Solyc10g006000.3	-1.01	0.50	0.00	0.00
Solyc08g066720.2	-1.05	0.48	0.00	0.02
Solyc05g009640.3	-1.10	0.47	0.00	0.01
Solyc11g010630.3	-1.11	0.46	0.00	0.01
Solyc08g067340.3	-1.13	0.46	0.00	0.00
Solyc04g005260.2	-1.14	0.45	0.00	0.04
Solyc08g006850.3	-1.16	0.45	0.00	0.00
Solyc06g065850.2	-1.20	0.44	0.00	0.00
Solyc03g119590.1	-1.29	0.41	0.00	0.00
Solyc01g090890.3	-1.38	0.38	0.00	0.00
Solyc03g005530.1	-1.41	0.38	0.00	0.00
Solyc03g095770.3	-1.41	0.38	0.00	0.00
Solyc10g085850.1	-1.47	0.36	0.00	0.00
Solyc01g014143.1	-1.54	0.34	0.00	0.00
Solyc05g008150.3	-1.63	0.32	0.00	0.00
Solyc02g086460.3	-1.78	0.29	0.00	0.00
Solyc04g015120.3	-1.85	0.28	0.00	0.00
Solyc12g006130.2	-2.16	0.22	0.00	0.00
Solyc09g007020.2	-2.17	0.22	0.00	0.00
Solyc03g098010.3	-2.78	0.15	0.00	0.00
